# Microglia centered pathogenesis in ALS: insights in cell interconnectivity

**DOI:** 10.3389/fncel.2014.00117

**Published:** 2014-05-22

**Authors:** Dora Brites, Ana R. Vaz

**Affiliations:** ^1^Research Institute for Medicines (iMed.ULisboa), Faculdade de Farmácia, Universidade de LisboaLisbon, Portugal; ^2^Department of Biochemistry and Human Biology, Faculdade de Farmácia, Universidade de LisboaLisbon, Portugal

**Keywords:** amyotrophic lateral sclerosis, microglia activation phenotypes, motor neuron, neuroinflammation, neurodegeneration, pathological cell–cell communication, SOD1G93A transgenic mouse/rat

## Abstract

Amyotrophic lateral sclerosis (ALS) is the most common and most aggressive form of adult motor neuron (MN) degeneration. The cause of the disease is still unknown, but some protein mutations have been linked to the pathological process. Loss of upper and lower MNs results in progressive muscle paralysis and ultimately death due to respiratory failure. Although initially thought to derive from the selective loss of MNs, the pathogenic concept of non-cell-autonomous disease has come to the forefront for the contribution of glial cells in ALS, in particular microglia. Recent studies suggest that microglia may have a protective effect on MN in an early stage. Conversely, activated microglia contribute and enhance MN death by secreting neurotoxic factors, and impaired microglial function at the end-stage may instead accelerate disease progression. However, the nature of microglial–neuronal interactions that lead to MN degeneration remains elusive. We review the contribution of the neurodegenerative network in ALS pathology, with a special focus on each glial cell type from data obtained in the transgenic SOD1G93A rodents, the most widely used model. We further discuss the diverse roles of neuroinflammation and microglia phenotypes in the modulation of ALS pathology. We provide information on the processes associated with dysfunctional cell–cell communication and summarize findings on pathological cross-talk between neurons and astroglia, and neurons and microglia, as well as on the spread of pathogenic factors. We also highlight the relevance of neurovascular disruption and exosome trafficking to ALS pathology. The harmful and beneficial influences of NG2 cells, oligodendrocytes and Schwann cells will be discussed as well. Insights into the complex intercellular perturbations underlying ALS, including target identification, will enhance our efforts to develop effective therapeutic approaches for preventing or reversing symptomatic progression of this devastating disease.

## INTRODUCTION

Amyotrophic lateral sclerosis (ALS) is a non-cell-autonomous disease targeting motor neurons (MNs) and neighboring glia, with microgliosis directly contributing to neurodegeneration (Beers et al., 2006; Lobsiger and Cleveland, 2007; Yamanaka et al., 2008a). Indeed, the neurodegenerative process in ALS was shown to be accompanied by a sustained inflammation in the brain and spinal cord (SC) (Bowerman et al., 2013). Recently, microglia were suggested to be implicated in ALS initiation (Gerber et al., 2012), as well as to lose their surveillance capacity by switching from an activated to a neurodegenerative phenotype as the disease progresses (Weydt et al., 2004; Dibaj et al., 2011). Therefore, a better therapeutic strategy should envisage the recovery of healthy microglia from those transformed cells near the most affected MNs in the SC. In such way we may preserve the passage of toxic mediators to environmental cells, and maintain the homeostatic conditions.

For the vast majority of patients with ALS, the etiology of the disorder is unknown. Actually, only some ALS cases (less than 10%) have been linked to mutations in a number of genes, including in the enzyme Cu, Zn superoxide dismutase 1 (SOD1), TAR DNA binding protein (TDP-43), fused in sarcoma (FUS), optineurin (OPTN), valosin-containing protein (VCP), ubiquilin 2 (UBQLN2), profilin 1 (PFN1), and chromosome 9 open reading frame 72 (C9ORF72) repeat expansions (Tovar et al., 2009a; DeJesus-Hernandez et al., 2011; Ince et al., 2011; Renton et al., 2011, 2014; Bertolin et al., 2013). Interestingly, both the non-genetic and the genetic forms of ALS are suggested to have common pathogenic mechanisms (Lilo et al., 2013), as well as similar clinical courses and dysfunctional features, such as the abnormal accumulation of neurofilaments in degenerating MNs (Julien, 2001). Actually, cytoplasmic aggregation of nuclear TDP-43 and FUS in the degenerating neurons and glia of ALS patients, and release of the accumulated cytoplasmic mutant SOD1 (mSOD1) to the extracellular space that can be taken up by other cells, are common features (Li et al., 2013; Ogawa and Furukawa, 2014). The identification of C9ORF72 repeat expansions in patients with ALS but without a family history of ALS challenged the division between genetic (familial) and non-genetic (sporadic) cases (Turner et al., 2013). As indicated by Kiernan (2014), the true substrate of ALS may reside in a pathogenic signature of nuclear protein mishandling.

There are several *in vitro* and *in vivo* models of MN degeneration. *In vitro* experimental models include SC cultures, NSC-34 cell line expressing the mSOD1 and organotypic cultures, while the axotomy-induced MN death, the naturally occurring ALS models, and the transgenic models are the most commonly used *in vivo* models (Elliott, 1999; Tovar et al., 2009a). Among the various transgenic models used in the study of ALS pathogenesis (Weydt et al., 2004; Kato, 2008), the transgenic rodent overexpressing mSOD1, in particular the SOD1G93A strain, is the most utilized and characterized. Transgenic mice containing other mSOD1 genes (G85R, G37R, D90A, or G93A missense mutations or truncated SOD1) and the related mutant (G86R) mouse have also shown progressive neurodegeneration of the motor system and resemblance to ALS (for review, see Van Den Bosch, 2011). Distinctive injurious effects between SOD1G93A and SOD1H46R on two different genetic backgrounds were recently recognized (Pan et al., 2012). Additionally developed models are based on TDP-43 (Wegorzewska et al., 2009; Liu et al., 2013; Yang et al., 2014) and FUS mutations (Verbeeck et al., 2012), but none of these models is currently used to study the pathogenesis of ALS and to test new drugs. Thus, the human mSOD1 murine model is the most widely used in the evaluation of the involved molecular targets, biomarkers and novel drugs/treatments for ALS. Apart from developing loss of MNs and symptoms that resemble human ALS by mSOD1, the model evidences molecular links between genetic and non-genetic cases of ALS (Andjus et al., 2009; Synofzik et al., 2010). To note, that non-genetic perturbations of the wild-type (wt) SOD1 protein may lead to SOD1 misfolding with a conformation much similar to genetic SOD1 variants (Cereda et al., 2006). Therefore, in this review we will summarize the most recent developments obtained in the SOD1G93A transgenic model to give consistency and cohesion between the data disclosed, and because we admit that common factors and pathways are shared in both genetic and non-genetic derived ALS cases, in particular changes in microglia performance and in neuron–glia communication.

It was initially considered that the selective death of MNs expressing the mutant protein was the player in the disease onset. However, non-cell-autonomous processes associated with mSOD1 in glial cells are believed to be implicated not only in disease progression and extent, but also to be related with the onset and early stage of the disease, thus underlying MN dysfunction and loss. Indeed, healthy glia evidenced to delay the progression of the disease (Boillée et al., 2006b; Yamanaka et al., 2008b) and the replacement of mSOD1 microglia by wt microglia slowed disease progression and prolonged mice survival (Lee et al., 2012). This finding is in line with previous studies showing that mSOD1 in microglia leads to the disease (Clement et al., 2003) and that the reduction of the mutant levels in the cells slows ALS progression (Boillée et al., 2006b). Indeed, damage to MNs by neighboring cells expressing mSOD1 seems to be required for MN degeneration (Pramatarova et al., 2001; Lino et al., 2002). Accumulating knowledge on the active participation of different microglia phenotypes in ALS was recently obtained when microglia were isolated from SOD1G93A rats at presymptomatic, symptom onset and end-stage periods (Nikodemova et al., 2013). Microglia were shown to be regionally different and to evidence a heterogeneity of phenotypes with the disease progression. Thus, it will be interesting to investigate the influence of changes caused by aging in the performance of the mutated microglia, namely on the interconnectivity with neurons and other glial cells. Moreover, our preliminary data indicate a decreased phagocytic and migration ability of healthy microglia by the soluble factors (SFs) released by NSC-34 cells expressing mSOD1, thus causing the loss of important microglia properties (Cunha, 2012). In addition, when this microglia was co-cultured with the mMNs we observed a reduction of the activation of matrix metalloproteinases (MMP)-2 and -9 in the extracellular media after cultivation for 4 days in vitro (DIV) (Barbosa, 2013), evidencing the beneficial effect of the healthy microglia in decreasing mMN stress.

Finally, we provide an outlook on the extent to which a diverse cellular environment may determine different pathological windows along disease progression opening new opportunities to explore distinct therapeutic approaches to either trigger a less reactive microglia phenotype at the early-onset, or a recovery of microglia dynamics at late-stage ALS. It will be also summarized recent advances in regenerative medicine technologies with potential to reverse or halt ALS progression by slowing MN death.

## NEURODEGENERATIVE NETWORKING IN ALS

Neuronal homeostasis and survival were shown to be compromised in ALS due to multiple aberrant biological processes and to deregulated communication between neurons and glial cells in the brain and in SC by the disease. ALS, once considered a MN disease, is now known to have multiple influences and regarded as a multi-cellular/multi-systemic disease (Nardo et al., 2011). In fact, MN death seems to be driven by a convergence of damaging mechanisms, including glial cell pathology and inflammatory conditions, such as microglial activation or the invasion of lymphocytes, and calcium dysregulation (Grosskreutz et al., 2010; Beers et al., 2011b). It can be also determined by excitotoxicity due to the selective loss of the astrocytic glutamate transporter GLT-1, and consequent accumulation of extracellular glutamate (Rothstein, 2009). Our late results with astrocytes isolated from SOD1G93A mice and cultivated for 13 DIV suggest that both GLT-1 and glutamate aspartate transporter (GLAST) are compromised (personal communication) and corroborate other findings indicating that expression of such transporters are less potently activated by lipopolysaccharide (LPS) in astrocytes from the mSOD1 model than in those from the wt mice (Benkler et al., 2013). Other contributing processes include from neurovascular changes and compromised barriers of the central nervous system (CNS), to dysfunctional communication between neurons, abnormal neuron–glia interactions, and microglia and astroglia loss-of-function.

In fact, for the optimal functioning of the CNS, i.e., brain and SC, accounts the constant immune surveillance promoted by cells such as microglia, and the blood–brain barrier (BBB), the blood–SC barrier (BSCB), and the blood–cerebrospinal fluid (BCSF) barrier that uniquely shield CNS from potential mediators of infection and damage. Recently, brain pericytes, as important components of the neurovascular unit in the CNS, have shown to have pleiotropic and regulatory activities in brain vessel function and homeostasis, blood flow and barrier function (Lange et al., 2012). Astrocytes also participate in maintaining homeostasis and supporting neuronal function. In addition to microglia, neurons have lately been indicated to intervene in immune responses through control of T cells infiltration into the CNS and glial cell immunoreactivity (Czirr and Wyss-Coray, 2012; Tian et al., 2012; Liblau et al., 2013). This integrative network sustains ionic, energetic, and redox homeostasis for proper function.

Moreover, ALS progression was suggested to involve cell-to-cell transmission of mSOD1 aggregates involving MNs, microglia and astrocytes, similarly to prion disease (Chia et al., 2010; Munch et al., 2011). Interestingly, the transcellular spread of SOD1 aggregates evidenced to not require cell-to-cell contacts but to depend from their fragmentation and extracellular release. However, additional studies should demonstrate that similar findings also occur *in vivo*. Intriguingly, while extracellular mSOD1G93A has shown to not have direct toxic effects on MNs, it morphologically and functionally activates microglia, supporting the non-cell-autonomous nature of MN toxicity in ALS (Zhao et al., 2010). Indeed, it was shown that this extracellular mSOD1 can be endocytosed into microglia, determining the activation of caspase-1 and the up-regulation of interleukin (IL)-1β (Zhao et al., 2013).

Another interesting concept is that astrocyte and microglia activation, which is regulated by a variety of signaling pathways, should not be considered merely as pernicious for CNS homeostasis, once it promotes metabolic support, wound healing and repair. In fact, the production of cytokines and chemokines initiate and coordinate diverse cellular and intercellular actions. Although neurons and glial cells of the CNS express receptors for cytokines and chemokines, the biological consequence of receptor activation is not fully understood. In contrast, it must be considered as part of the pathological processes the excessive or deregulated signaling pathways leading to microglia and astrocyte abnormalities that culminate in abnormal CNS function.

Thus, progressive neurodegeneration of MNs in ALS may result from a combination of intrinsic MN vulnerability to mSOD1 aggregates and of non-cell-autonomous toxicity derived from neighboring cells (**Tables 1** and **2**). Therefore, pathological changes in ALS indicate a broken homeostasis in the CNS. We will focus on how CNS homeostasis is lost in ALS and in what way BBB and neural networks dysregulation contribute to neurodegeneration in ALS.

**Table 1:** Functional alterations of motor neurons (MNs) in amyotrophic lateral sclerosis (ALS): candidate molecular targets.

Changes in MN signaling by ALS	ALS stages	Reference
Elevation of matrix metalloproteinase (MMP)-9	Pre-symptomatic phase	Soon et al. (2010), Kaplan et al. (2014)
Impaired antioxidative Keap1/Nrf2/ARE system	Along disease progression	Mimoto et al. (2012)
Oxidative and nitrosative stress	Early phase	Drechsel et al. (2012)
Glutamate excitotoxicity	Onset and progression	Spalloni et al. (2013)
Release of ATP	Along disease progression	Glass et al. (2010)
Protein misfolding, aggregation, and accumulation	Onset and progression	Julien (2001), Li et al. (2013)
Changes in fractalkine (CX3CL1), CD200, and CCL21	Not clarified	–
Decreased high-mobility group box protein 1 (HMGB1) cellular expression	Advanced phase	Lo Coco et al. (2007), Fang et al. (2012)
Release of neuregulin-1 (NRG1)	Along disease progression	Song et al. (2012)
Up-regulation of major histocompatibility complex (MHC) class I and β2-microglobulin mRNAs	Along disease progression	Staats et al. (2013)

**Table 2:** Glial impairment and deregulated glia–motor neuron (MN) interconnectivity in amyotrophic lateral sclerosis (ALS).

Mutant SOD1 cells	Loss of supportive functions	Contribution to ALS disease and MN death	Reference
Astrocytes	Deficient astrocyte-specific glutamate transporter EAAT2 (GLT-1)	Increase in the excitatory amino acid glutamate	Valori et al. (2014)
	Increased release of D-serine	co-activator of the *N*-methyl-D-aspartate (NMDA) receptors, exacerbating glutamate toxicity on MNs	Valori et al. (2014)
	Mitochondrial dysfunction	Increased production of reactive oxygen species (ROS)	Valori et al. (2014)
	Release of interferon-γ and transforming growth factor-β (TGF-β)	Increased neuroinflammation	Valori et al. (2014)
	Ubiquitin- and active caspase-3-immunopositive	Degenerating astrocytes at the pre-symptomatic stage when MNs show axonal damage but are still alive	Valori et al. (2014)
	Increased nerve growth factor (NGF) and NO production	MN apoptosis	Pehar et al. (2004)
Astrocytes (aberrant)	Increased S100B and connexin-43 (Cx-43)	Decreased MN survival	Diaz-Amarilla et al. (2011)
Microglia (spinal cord – early stage)	Recruitment of peripheral monocytes to the CNS	Neuronal viability impairment	Butovsky et al. (2012)
Microglia (spinal cord – end stage)	Decreased expression of M1 and M2 markers	Decreased reactivity to stimuli	Nikodemova et al. (2013)
Microglia (M2) – early stage	High levels of anti-inflammatory cytokines and neurotrophins	Enhancement of MN survival (neuroprotection) at ALS early stage	Zhao et al. (2013)
Microglia (M1) – progressive stage	Increased release of reactive oxygen species (ROS), tumor necrosis factor-α (TNF-α) and interleukin (IL)-1β	Toxicity to MN (death) in the late rapid phase of ALS	Zhao et al. (2013)
Dystrophic microglia – end stage	Decreased migration and phagocytosis by aging (not yet confirmed in ALS)	Neuronal degeneration by failure of the senescent microglia response to stimuli	Luo and Chen (2012)
Oligodendrocytes	Loss of the monocarboxylate transporter 1 (MCT1)	Decreased delivery of the metabolic substrate lactate to MNs and axonal sufferance	Philips et al. (2013)
NG2^+^ cells	Increased proliferation rate and degeneration of early-born oligodendrocytes	Gray matter demyelination	Kang et al. (2013)
Schwann cells	Signs of distress at the asymptomatic stage	Not known	Valori et al. (2014)

### MOTOR NEURON DYSFUNCTION

A certain number of cases are linked to mutations in SOD1 and candidate mechanisms are the formation of protein aggregates and pro-oxidant effects. Therefore, most of the experiments are generally conducted in the MN-like hybridoma cell line NSC-34 expressing human mSOD1 (hSOD1G93A) (Tovar et al., 2009a) or in the transgenic mice generated by Gurney et al. (1994) that also over-express hSOD1G93A (Riboldi et al., 2011).

SOD1 aggregates are observed in both genetic and non-genetic ALS cases, but their contribution to MN toxicity remains to be established, although deregulation of Golgi, endoplasmic reticulum (ER), and mitochondria, together with axonal transport defects have been indicated (for review, see Boillée et al., 2006a; Rothstein, 2009). Interestingly, when working with the NSC-34/hSOD1G93A cells we found that one of the most sensitive indicators of SOD1 accumulation and neuronal dysfunction was the elevation of MMP-9, but not of MMP-2 that remained unchanged (Cunha, 2012; Vaz et al., 2014). Elevation of the MMP-9 levels was previously observed in the SC of ALS mice from pre-symptomatic phase, predominantly in MNs but also in glia (Soon et al., 2010). These authors suggest that circulating MMP-9 is associated with the disease onset and is mainly derived from degenerating SC MNs and circulating cells. Its inhibition was shown to enhance animal survival in more than 30% (for review, see Riboldi et al., 2011). It was also found in the CNS, muscles, plasma, and skin of patients. Major inducers are reactive oxygen species (ROS) and cytokines released by microglia. Once MMP-9 is indicated to promote the regeneration of the injured neuron, it may be hypothesized that its increase results from a MN response to the pathology. However, it was recently claimed that MMP-9 is indeed a determinant of the selective neurodegeneration (Kaplan et al., 2014). The Authors demonstrated that MMP-9 is only expressed by the selectively vulnerable fast MNs and can originate ER stress and axonal dye-back. This finding provides a basis for considering MMP-9 as a candidate target for novel therapeutic approaches to ALS.

Oxidative damage at the level of proteins, lipids, and DNA was also observed in the transgenic hSOD1 mice models (Liu et al., 1999; Casoni et al., 2005; Poon et al., 2005; Barbosa, 2013) where mSOD1 revealed to be the most severely oxidized protein mouse (Andrus et al., 1998). Also accounting to MN disturbance is the failure of the Keap1/Nrf2/ARE system in regulating stress proteins, as evidenced along disease progression in MNs from the SC of hSOD1G93A mice (Mimoto et al., 2012). In addition, failure in MN autophagy was revealed as critical in the pathogenesis and progression of ALS (for review, see Pasquali et al., 2009; Chen et al., 2012; Otomo et al., 2012).

Currently, there is no cure for the ALS disease, although Riluzole, the only drug approved by the U.S. Food and Drug Administration (FDA), was shown to prolong median patient survival by 2–3 months and to be more effective when administered at an early stage of the disease (Zoccolella et al., 2007; Lee et al., 2013a). More potent therapeutic strategies may derive from better target identification and clarification of signaling mechanisms able to be modulated and from the use of cell-based therapies, such as the administration of mononuclear cells from human umbilical cord blood (Garbuzova-Davis et al., 2012) and of mesenchymal stromal (stem) cells (Uccelli et al., 2012), as referred to in Section “Challenges to Nerve Regeneration in ALS.” Bellow we introduce what is known about the most prominent MN membrane proteins and SFs suggested to be implicated in ALS pathology, but still deserving to be more explored.

#### Perturbations in glutamate handling

Glutamate excitotoxicity is one primarily cause of neuronal death by necrosis and apoptosis and a critical player in ALS onset and progression. Indeed the blockade of the glutamate transporter in a SC organotypic slice model from SOD1G93A rats has shown to result in an increased survival of MNs (Yin and Weiss, 2012). Glutamate, by activating the glutamatergic ionotropic receptor *N*-methyl-D-aspartate (NMDAR), triggers the influx of Ca^2^^+^ into neurons, increasing its intracellular levels (for review, see Spalloni et al., 2013). In these conditions, changes in mitochondria dynamic properties are produced leading to excessive oxidative phosphorylation and increased generation of ROS and reactive nitrogen species (RNS) that culminate in apoptosis. In parallel, the NMDAR excitotoxicity also results in the release of Ca^2^^+^ from the ER. In addition, it was shown that ER stress resulting from the accumulation of aggregated mSOD1 and dysfunction of the unfolded protein response (UPR) activation contributes to the apoptotic signaling cascade in ALS (for review, see Sofroniew, 2009).

Besides the synaptic glutamate pool directly implicated in the excitatory neurotransmission it has been lately additionally considered the extra-synaptic glutamate pool that influences cell communication. This pool, mainly derived from astrocyte and microglia release, is greatly increased by pathological stimuli (for review, see Rodriguez et al., 2013). Besides being implicated in many physiological conditions glutamate contribution to neuron–glia alterations of homeostasis and to the pathophysiology of neurodegenerative diseases, such as ALS, needs to be explored in the near future, due to controversial results. In fact, although linkage between glutamate and neuroinflammation was suggested to have a role in potentiating MN death in a model mimicking ALS disease (Tolosa et al., 2011), other Authors have demonstrated that glutamate by itself was not able to induce MN damage in ALS (Tovar et al., 2009b). Indeed, it has been questioned the involvement of glutamate in ALS-induced MN death (Le Verche et al., 2011) and the cytotoxicity of the cerebrospinal fluid (CSF) from patients with ALS evidenced to not be related to glutamate (Gomez-Pinedo et al., 2013).

#### Membrane-bound and soluble fractalkine (CX3CL1)

Fractalkine (FKN) mRNA is dominantly expressed in neuronal cells, particularly in those at the cortex, hippocampus, caudate putamen, thalamus, and olfactory bulb (Harrison et al., 1998). FKN mRNA was also detected in unstimulated astrocytes (more) and microglia (less) (Mizuno et al., 2003). Interestingly, FKN immunoreactivity and mRNA was observed also in the rat SC and dorsal root ganglia neurons, but not in glia, and levels were not enhanced by neuropathic conditions (Verge et al., 2004). FKN, or CX3CL1, exists as a membrane-bound and soluble protein (sFKN) allowing both adhesive and chemoattractive properties (Bazan et al., 1997). In fact, the sFKN has potent chemoattractant activity, recruiting CXCR1-expressing T cells, monocytes, and microglia to the injured neurons, as well as in regulating the phagocytic capacity of microglia (Cardona et al., 2006). sFKN was shown to increase upon stimulation of glutamate (Chapman et al., 2000) and to induce proliferation of human microglia (Hatori et al., 2002). The constitutive expression of FKN and its receptor CX3CR1 in microglia indicates its involvement in fundamental processes of communication between neurons and microglia (Harrison et al., 1998).

Under stimulation, such as an excitotoxic stimulus (Chapman et al., 2000), the membrane-bound form of FKN is rapidly cleaved from cultured neurons and significantly reduces neuronal NMDA-induced apoptosis (Deiva et al., 2004). Indeed attenuated glutamate-induced neuronal cell death was observed after treatment of primary neuron–microglia co-cultures with sFKN (Noda et al., 2011). Proteolytic cleavage of CX3CL1 is mediated by action of metalloproteinase ADAM10 (Gough et al., 2004), ADAM17 (Garton et al., 2001; Tsou et al., 2001), cathepsin S (CatS) (Clark et al., 2009), and MMP-2 (Bourd-Boittin et al., 2009). Thus, cleavage of FKN may be prevented by the inhibition of MMPs (Chapman et al., 2000). The source of CatS is the activated microglial cells that upon stimulation with LPS secrete sFKN to the extracellular media (Clark et al., 2009). Similarly, stromal cell-derived factor-1 (SDF-1) was indicated to stimulate the expression of ADAM17 and to increase sFKN, while up-regulating FKN expression (Cook et al., 2010).

In addition, FKN has a neuroprotective function by inhibiting the nitric oxide (NO) production and the expression of inducible NO synthase (iNOS) mRNA in activated microglia (Mizuno et al., 2003). Interestingly, it was observed that FKN expression was reduced in the brain of aged rats probably accounting for the increase in microglial activation in such condition. In fact, treatment with FKN has attenuated the age-related increase in microglial activation (Lyons et al., 2009).

Taken together, FKN seems to have both intrinsic and anti-inflammatory properties in the CNS and to act by interfering with toxic microglial–neuron interactions (Suzuki et al., 2011). Whether FKN may have a role in the development of ALS is still not known.

#### High-mobility group box 1 protein

**FIGURE 1:**
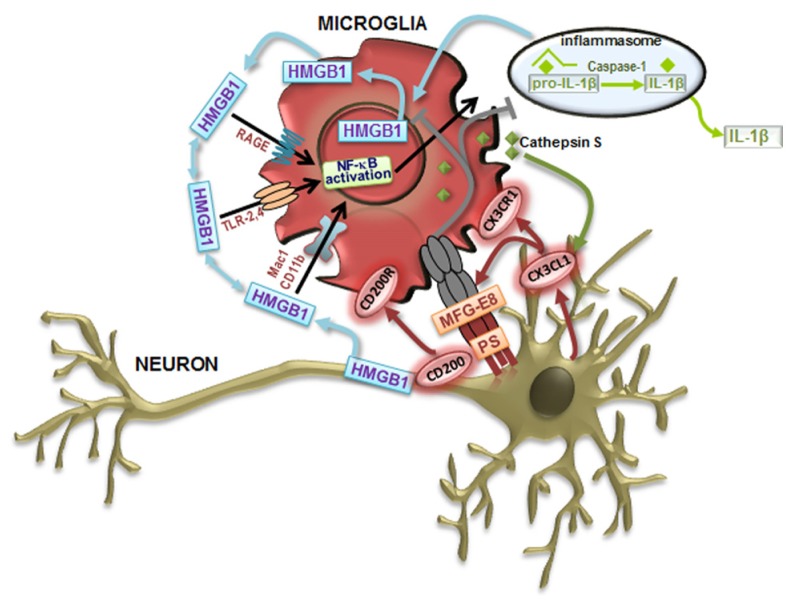
**Neuron–microglia communication signaling pathways that modulate microglia cell phenotypes.** Toll-like receptor (TLR) signaling contributes to classically activated microglia (M1) in response to damage-associated molecular patterns (DAMPs). Following recognition of DAMPs, TLRs activate downstream signaling cascades, activate nuclear factor-κB (NF-κB) inducing the transcription of inflammatory mediators associated with the M1-like microglial cell phenotype, such as the proinflammatory cytokines interleukin (IL)-1β and tumor necrosis factor (TNF)-α. The production of IL-1β can be achieved by the inflammasome activation by DAMPs, leading to caspase-1 activation that orchestrates the cleavage of pro-IL-1β to form active IL-1β, which leaves and binds to the IL-1 receptor, resulting in inflammation. Following activation, microglial cells augment the immune response by releasing metalloproteinases (MMPs) and increasing proinflammatory cytokines. High-mobility group box 1 (HMGB1) is an alarmin that signals cell damage in response to injury and are associated to all the described signaling events. HMGB1 released by activated microglia and damaged neurons concur for a vicious cycle mediating chronic, progressive neurodegeneration associated with neuroinflammation. HMGB1 can interact with receptors that include RAGE (receptor for advanced glycation end products), TLR-2, TLR-4, Mac-1 also known as CD11b, and possibly others. Alternatively activated microglia (M2) functions include phagocytosis. Milk fat globule factor-E8 (MFG-E8) produced by microglia recognizes phosphatidylserine (PS) as “eat me” signals expressed on the surface of apoptotic neurons, triggering a signaling cascade that stimulates phagocytosis to engulf the dying cell. In order to maintain a quiescent microglia phenotype (M0) under steady-state conditions, neurons suppress the activation of microglia through cell–cell contact (CD200–CD200R) and by the release of the chemokine ligand 1 (CX3CL1) mediated by the cathepsin S that binds to its receptor CX3CR1 on microglia.

High-mobility group box 1 (HMGB1) protein, also known as amphoterin, is an inflammatory factor that can be released by astrocytes, microglia, and neurons, mainly when cells are dying (for review, see Fang et al., 2012). HMGB1 has dual activities depending on whether is alone (probably promoting inflammation resolution and tissue regeneration) or forming complexes with several proinflammatory mediators (potentiating inflammation and promoting innate immune cell activation) (Bianchi, 2009). Nuclear HMGB1 regulates transcription of different sets of genes, including proinflammatory genes (Bianchi and Manfredi, 2009; Park et al., 2009; Wong, 2013). Beneficial effects were observed in early CNS development but increased levels of HMGB1 were shown to be correlated with apoptosis and degeneration of neurons (Liu et al., 2009; Kawabata et al., 2010). When neurons are injured, secretion of HMBG1 activates microglia through receptor for advanced glycation end products (RAGE), Toll-like receptors (TLRs) 2, 4, and 9, as well as Mac1 receptors (Kim et al., 2006a; Park et al., 2006; Neusch et al., 2007), as depicted in **Figure 1**. Release of inflammatory mediators by activated microglia further induces neuronal necrosis. HMGB1 was indicated to decrease in neurons and to increase in astrocytes with aging (Enokido et al., 2008). When expression and localization of HMBG1 was evaluated in the lumbar SC of SOD1G93A transgenic mice, although intense reactivity was found, no differences were obtained between controls and the SOD1 mice (Lo Coco et al., 2007). However, since HMBG1 was identified in the cytoplasm of astrocytes and microglia in SC samples from ALS patients (Casula et al., 2011) it may trigger TLR signaling pathways. The finding was observed at the late ALS phase and, thus, it will be important to follow the TLR/RAGE cascade in animal models at different stages of the disease. In contrast, a progressive reduction of HMGB1 immunopositive MNs was found at advanced stages and may reflect the loss of MNs, reduced synthesis or enhanced released of the cytokine (Lo Coco et al., 2007). Additional studies are needed to investigate the causative hypothesis indicated for the decreased HMGB1 immunoreactivity in MNs, inasmuch since it can also have beneficial effects on neuroregeneration (Fang et al., 2012).

#### CCL21

CCL21 was shown to be implicated in signaling neuronal injury to microglia through the receptor CXCR3. CCL21 expression was demonstrated to increase in cortical neurons, *in vitro*, 2 h after excitotoxic stimulus (de Jong et al., 2005). Intriguingly, these authors demonstrated its location within vesicles that are transported along the neuronal process till presynaptic structures. This chemokine is considered a chemotactic agent important to drive microglia to the site of lesion. Astrocytes were also indicated to have the receptor CXCR3, but unless high levels of CCL21 are produced, no relevant changes can be observed, indicating separate functions from microglia (van Weering et al., 2010). CCL21 expression in CNS revealed to induce a massive brain inflammation, but not lymphocytic infiltration in transgenic mice expressing the chemokine (Chen et al., 2002). Interestingly, CCL21/CXCR3 signaling axis was never explored in ALS.

#### CD200

The membrane glycoprotein CD200 is expressed in neurons and in endothelial cells and its receptor CD200R is restricted to cells of myeloid origin including macrophages and microglia. CD200 was also evidenced to be induced by kainic acid in microglia (Yi et al., 2012) and the authors have suggested that microglia are maintained in an activated state with autocrine signaling by interactions between microglial CD200 and CD200R, as well as in a surveillant/quiescent state by interactions between neuronal CD200 and microglial CD200R. In addition, they also demonstrated that IL-4 leads to an increased expression of CD200 what may be the mechanism down-regulating microglia activation whenever IL-4 is produced. In conclusion, deficits in CD200–CD200R system exacerbate microglia activation and the release of proinflammatory cytokines (for review, see Jurgens and Johnson, 2012). Curiously, multivariate analyses of gene expression have previously identified alterations on the CD200R expression in presymptomatic SOD1 mice model (Chen et al., 2004). Thus, more studies on the role of CD200/CD200R in ALS are needed to evaluate whether its disruption is implicated in ALS.

#### Neuregulins

Neuregulins have been showing to be implicated in a wide range of neurological and psychiatric disorders including multiple sclerosis, schizophrenia, and AD. Neuregulin precursors are expressed predominantly in cortical neurons, but also accumulate at the surface of white matter astrocytes. CSF neuregulin was found reduced in ALS and increased in AD (Pankonin et al., 2009). Neuregulin-1 (NRG1) is a growth and differentiation factor that binds to erbB receptors in microglia. NRG1–erbB signaling is activated after peripheral nerve injury and contributes to microgliosis and neuropathic pain (Calvo et al., 2010). Recently, it was suggested that NRG1 released from damaged neurons and other cells in the SC triggers microglial activation leading to progressive MN degeneration in ALS (Song et al., 2012). Indeed, it was observed that in the early stage of ALS there was activation of NRG1 receptors on microglia. Later on, a reduced membrane-bound NRG1 and an increased mRNA expression were noticed in the SC. More studies are, however, needed to sustain the NRG1 contribution to ALS pathogenesis.

#### β2-Microglobulin

Major histocompatibility complex (MHC) class I and class II molecules present fragments of peptide antigens to CD8^+^ or CD4^+^ T cells, respectively, and cells lacking such molecules are unable to interact with immunocompetent T cells (Parnes, 1989). Presentation of antigenic peptides to CD8^+^ cells is mediated by β2-microglobulin, which is non-covalently bound to MHC class I. Despite being recently observed during hippocampal neurodevelopment (Leinster et al., 2013), adult neurons do not constitutively express MHC class I (Arthur-Farraj et al., 2012). In contrast, spinal MNs were shown to display a high constitutive expression of both MHC class I and β2-microglobulin mRNAs (Thams et al., 2009). A latest paper describes a strong up-regulation of β2-microglobulin in MNs during disease progression in the SOD1G93A mice model, which revealed to be important for the ALS mouse survival (Staats et al., 2013). Therefore, β2-microglobulin target driven therapies may be of help in strategies counteracting ALS.

### GLIAL CELL RESPONSES

It is believed that non-neuronal cells expressing mSOD1 may secrete toxic mediators or fail to secrete trophic factors, or both, resulting in either reduced function or survival of MNs. Indeed, several SFs were shown to drive axonal degeneration and regional-specific microglia activation expressing SODG93A was suggested to be implicated (Kim et al., 2006b). This calls our attention for the existence of multiple factors, and MNs and glial cells interplay in contributing to ALS onset and progression. Microglia activation and dysfunction have lately been related with the onset, but mostly with the progression, of several neurodegenerative diseases. Particularly, in ALS, it has been suggested that besides MNs, also glia and muscles are implicated in the disease.

Prominent neuroinflammation is a pathological hallmark in human ALS and mouse models of the disease. Gliosis and accumulation of large numbers of activated microglia and astrocytes can be observed in CNS and in SC areas. Active contributions of glial cells in ALS pathology have recently been reviewed (Lasiene and Yamanaka, 2011; Zhao et al., 2013). Conflicting results were published on whether the expression of mSOD1 in astrocytes and microglia really contributes to the progression of ALS disease. Some argues against (Yoshii et al., 2011) and others in favor (for review, see Boillée et al., 2006b). Overall, it will be important to identify the molecules released by glia, as well as the ones secreted by dysfunctional neurons targeting microglia or even acting retrogradely.

#### Toll-like receptor activation

Glial cells (astrocytes, microglia, oligodendrocytes, and Schwann cells), as well as neurons are known to express different members of the TLR family (Lee et al., 2013b). Increasing evidence indicates that, in the absence of pathogens, TLR signaling can be activated by molecules released by the injured tissue, namely the HMGB1 protein, one of the damage-associated molecular patterns (DAMPs) molecules (Bianchi and Manfredi, 2009). Recent findings have underlined the activation of TLRs and RAGE signaling pathways in ALS (Casula et al., 2011). TLR2, TLR4, and RAGE expression was increased in reactive glial cells in both gray (ventral horn) and white matter in SC. TLR2 was predominantly detected in cells of the microglia/macrophage lineage, whereas TLR4 and RAGE were strongly expressed in astrocytes. Activated macrophages (He et al., 2012) and microglia (personal communication) release HMBG1 that promotes the secretion of IL-1β and IL-18 further inducing the necrosis of neighboring neurons and the amount of extracellular HMBG1, which binds to microglial Mac1 leading to the activation of nuclear factor-κB (NF-κB) pathway (Gao et al., 2011) and inflammasome (Lu et al., 2013), forming a vicious circle that sustains progressive neurodegeneration (**Figure 1**). Recent data have demonstrated that NF-κB activation in wt microglia causes MN death providing a therapeutic target for ALS (Frakes et al., 2014).

When evaluating whether extracellular mSOD1 caused a direct or indirect injury to MNs, it was observed that neurodegeneration was mediated by microglia and concerted activation of CD14/TLR pathway involving both TLR2 and TLR4 (Zhao et al., 2010). In addition, granulocyte macrophage-colony stimulating factor (GM-CSF), a pleiotropic cytokine predominantly released by astrocytes that up-regulates TLR4 and CD14 expression in microglia, may function to exacerbate TLR signaling in ALS disease (Parajuli et al., 2012). Indeed, when GM-CSF was blocked it was observed a delayed onset and increased life span in the ALS mice (Turner and Talbot, 2008). The activation of these pathways may contribute to the progression of inflammation and to a further injury to MNs.

#### NG2 Cells, oligodendrocytes, and Schwann cells

Oligodendrocytes in CNS, and Schwann cells in the peripheral nervous system (PNS), are responsible for the myelin sheaths surrounding neurons which provide electrical insulation essential for rapid signal conduction. Schwann cells also participate in the clearance of debris and in guiding the axon after neuron damage (Ilieva et al., 2009). In the SC injury, reactive gliosis emerges in the lesion accompanied by the up-regulation of chondroitin sulfate proteoglycans (CSPGs) in oligodendrocytes and Schwann cells.

Considering the regenerative capacity of NG2 cells, one of the first cells responding to any alteration in CNS environment, it will be important to clarify their role in ALS since they can be mobilized to originate cells or to release factors. Nevertheless, NG2 cells were shown to remain committed to an oligodendrocyte lineage in both controls and mutant ALS mice, indicating their scarce participation (Kang et al., 2010). However, increased proliferation rate of NG2 revealed to mediate an elevated number of early-born oligodendrocytes that degenerate, resulting in gray matter demyelination in ALS mice and human CNS (Kang et al., 2013).

Although only a few studies have examined whether oligodendrocytes or myelin sheaths have a role in ALS, the myelin abnormalities consisting in loss of compact myelin and lamellae detachment in the SC of pre-symptomatic SOD1 transgenic rats and aggravated at symptomatic stages (Lasiene and Yamanaka, 2011) suggest that it may be an interesting target for further study. Biochemical examinations of the myelin structural components revealed a decrease in the phospholipid content along disease progression, as well as in cholesterol (already in the early presymptomatic stage) and cerebrosides (in the paralyzed animals) (Niebroj-Dobosz et al., 2007). Degeneration of oligodendrocytes was observed in human patients and mice models of ALS prior to the disease (Kim et al., 2013). Cells that were lost were replaced by newly differentiated oligodendrocyte precursor cells, which, however, evidenced reduced myelin basic protein. This finding is suggestive that they may contribute to MN degeneration in ALS (Philips et al., 2013). Moreover, these cells evidenced a loss of the monocarboxylate transporter 1 (MCT1), thus compromising the supply of lactate to MNs (**Table 2**).

Little is known about Schwann cell involvement in ALS pathology. Schwann cells are located near the MN axons and are known to bridge between denervated and reinnervated endplates, and to guide axonal sprouts (Magrane et al., 2009). Expression of mSOD1 in perisynaptic Schwann cells was suggested to interfere with the trophic maintenance of normal or regenerating motor axons (Inoue et al., 2003). *In vivo* evidence suggests that glial fibrillary acidic protein (GFAP) up-regulation in the stressed/proliferating Schwann cells may be the underlying pathological events (Keller et al., 2009). Conversely, different results were obtained in SOD1G73R mice where elimination of mutant SOD1G37R from Schwann cells failed to slow disease progression (Lobsiger et al., 2009). Nevertheless, Wang et al. (2012) found that knockdown of mSOD1 in Schwann cells of SODG85R transgenic mice delayed disease onset and extended survival indicating that SOD1G85R expression is neurotoxic. These results imply that diverse mutations confer different outcomes to cell toxicity and, in the case of Schwann cells, oxidative damage seems to be an important feature in the context of ALS.

#### Astrocyte reactivity

Astrocytes are a potential source of both pro- and anti-inflammatory cytokines and are ideally placed in close proximity to BBB and BSCB, thus translating signals from the periphery to the CNS. Although not being immune cells, they can also contribute to the immune response. Astroglial activation, or astrogliosis, is characterized by hyperplasia, hypertrophy of cell bodies and cytoplasmic processes, up-regulation of intermediate filament proteins, namely GFAP and vimentin, mediating a histologically apparent glial scar at the lesion site in the damaged SC (Sofroniew, 2009). Reactive astrogliosis in ALS was first revealed in 1990s by increased GFAP staining in the subcortical white matter (Kushner et al., 1991), and later on similarly observed in the ventral and dorsal horns of the SC, as well as in the transition between gray matter and anterior and lateral funiculi, where the dystrophy of neuritis exists (Schiffer et al., 1996). SC astrocytes were shown to assume a neurotoxic phenotype in response to extracellular ATP. In SOD1G93A astrocytes this activation is mediated through P2X7 receptor signaling (Gandelman et al., 2010). Reactive astrocytes surround degenerating MNs in patients and transgenic animal models of ALS, and in particular those localized in the ventral SC of SOD1G93A mice, are a source of nerve growth factor (NGF) and NO, which are both required for MN apoptosis (**Table 2**) (Pehar et al., 2004). Interestingly, the NO-induced MN death was shown to be mediated by astrocytes expressing SOD1 and TDP-43 mutations (Rojas et al., 2014). Ferraiuolo et al. (2011) have shown that the increase in total NGF is due to the secreted pro-NGF fraction before cleavage to the mature form, achieving a twofold increased ratio in the SOD1G93 mice as compared to control conditions. Such finding may derive from the increased ALS-associated MMP-9 (Niebroj-Dobosz et al., 2010), one of the enzymes that degrade mature NGF in the extracellular space. In this scenario, neuroprotection of MNs could be achieved by promoting pro-NGF cleavage.

Recently, it was demonstrated that loss of GFAP did not affect disease onset and marginally shorten the SOD1H46R mice survival, indicating that GFAP only plays modulatory effects (Yoshii et al., 2011), at least in this mice. On the contrary, astrogliosis was observed in the SOD1G93A mice in either symptomatic or presymptomatic phase, preceding microglial activation (Yang et al., 2011). Indeed, depending on the ALS animal model used, astrocyte activation was observed to occur earlier or later and to be rather complex, increasing and decreasing in waves at different times throughout the disease (for review, see Evans et al., 2013). Despite no differences in the astrocyte number, increased GFAP labeling in the ventral horn of lumbar SC from SOD1G93A mice was observed before MN loss (Gerber et al., 2012). Astrocytes derived from such mice evidenced to uptake glutamate less efficiently and showed a reduced trophic response to activation, deficiently protecting MNs (Benkler et al., 2013). Attesting this low astrocyte efficiency, the mainly expressed astroglial S100B protein was found to be decreased in CSF (Sussmuth et al., 2003) and serum (Otto et al., 1998) from ALS patients. Interestingly, exposure of primary cultures of astrocytes from ALS patients to CSF evidenced to enhance GFAP and S100B expression (Shobha et al., 2010). Similarly to neurons, astrocytes have demonstrated constitutive and regulated expression of FKN, which may control the migration and function of the microglia (Sunnemark et al., 2005). Additional contribution of astrocytes to MN death involves the release of ROS mediated by mitochondrial dysfunction and of D-serine that is a co-activator of the NMDARs, thus exacerbating glutamate excitotoxicity, among other factors as indicated in **Table 2** and reviewed in Valori et al. (2014).

A recent publication has characterized a specific astrocytic phenotype (aberrant astrocytes) obtained from primary SC cultures of SOD1G93A symptomatic rats (Diaz-Amarilla et al., 2011). These aberrant astrocytes isolated on the basis of marked proliferative capacity and lack of replicative senescence, lack GLT-1 and the NG2 marker and were shown to release toxic factors accounting for a MN hostile environment. This specific astrocyte phenotype expressing increased S100B and connexin-43 (Cx-43) is abundant in the symptomatic phase of the disease and seems to be located close to MNs, representing a new potential target for delaying ALS progression.

Data in *postmortem* tissues of ALS patients revealed changes in the morphology of astrocytes together with elevated GFAP and aldehyde dehydrogenase family 1, member L1 (ALDH1L1) (Philips and Robberecht, 2011), corroborating findings in animal models of ALS. Indeed, mSOD1 gene excision from microglia and selective reduction in astrocytes significantly slowed disease progression (Yamanaka et al., 2008b). However, there is still some controversy on whether astrogliosis is detrimental or beneficial, what surely will depend from spectrum intensity and toxicity potential of the aberrant phenotype.

#### Microglia activation

Microglia are the immune resident cells of the CNS with both a supporting role to neurons and astrocytes, and immunological properties with either neuroprotective or neurotoxic potential. Though microglia activation was indicated to precede astrocyte reactivity (Alexianu et al., 2001), reduced neuroprotective behavior of mSOD1 microglia in a resting-state (Sargsyan et al., 2011), and at the disease end-stage (Nikodemova et al., 2013) was documented. If confirmed, boosting of microglia with stimulatory factors may reveal to be clinically useful.

In a recent paper, Roberts et al. (2013) verified that incubation of microglia with aggregated SOD1 first drives its location at the membrane level, and later within the cell. Moreover, supernatants of these SOD1 activated microglia caused a significant decrease in MN viability, which was not related with tumor necrosis factor-α (TNF-α) secretion, NO, or superoxide anion radical. The toxic factors involved are currently unknown, although it was shown that expression of mSOD1 increases TNF-α secretion (Liu et al., 2009), which may induce neurotoxicity by increasing the glutamate release by microglia in an autocrine manner (Takeuchi et al., 2006). Therefore, a very early stage of the disease may rely in this link between protein aggregation and microglial activation. Indeed, neurotoxic potential of mutant microglia in MN degeneration in ALS is a very well documented issue: (1) mSOD1 acting on microglia is required to cause the disease (Clement et al., 2003); (2) limiting mutant damage to microglia slows progression (Boillée et al., 2006b); replacement of mSOD1 microglia for wt microglia delays disease and prolongs mice survival (Lee et al., 2012).

Microgliosis at sites of MN injury is a neuropathological hallmark of ALS and recent therapeutic interventions are looking into factors capable of skewing microglia neurotoxic potential into a neuroprotective phenotype. However, a trophic role for activated microglia has been suggested at early stages of the disease (for review, see Lewis et al., 2012). In the SOD1G93A mice, it was observed a decrease in microglia number in the entire SC at the pre-symptomatic age (Gerber et al., 2012). The authors identified two diverse microglia subpopulations (low and high Iba1 expression cells), suggesting a distinct microglia involvement at pre- and early-symptomatic ALS stages. Interestingly, microglia heterogeneity observed between cervical and lumbar SC regions in ALS mice may derive from different environmental specificities determined by local MN/astrocyte/lymphocyte disposition and activation (Beers et al., 2011b). While cortical microglia appear unaffected by the disease, additional studies evidenced an increased microglial number in the lumbar SC at symptom onset, and neither the typical inflammatory nor the anti-inflammatory phenotypes were identified at end-stage (Nikodemova et al., 2013). In addition, elevated TNF-α gene expression and immunoreactivity were observed in lumbar SC of mSOD1 mice and related with invading microglia (Yoshihara et al., 2002). Early microglia activation in ALS may be further explored *in vivo* by positron emission tomography (PET). The possibilities of PET suggest its valuable contribution to monitor the progression of the disease and the efficacy of the therapy in use (Corcia et al., 2012).

***Context-dependent neuroprotective and neurotoxic properties***. An important neuroprotective role of microglia is phagocytosis. Following signaling by neuronal FKN, milk-fat globule EGF factor-8 protein (MFG-E8, SED1) is up-regulated and serve as a bridge via specific integrins between apoptotic neurons and microglia (**Figure 1**) (Leonardi-Essmann et al., 2005). The authors hypothesize that MFG-E8 is assembled on the surface of exosomes and apoptotic neurons so microglia can recognize their target cells. MFG-E8 seems to be essential for microglia engulfment and removal of the dying neurons. An interesting concept is that inflammatory microglia can also phagocyte viable neurons (Fricker et al., 2012a) through MFG-E8 mediation (Fricker et al., 2012b) accounting for a reduced number of neurons. Phagocytic ability may, however, be lost if the cell is continuously stressed by a neurotoxic stimulus.

Our recent data with the neurotoxic unconjugated bilirubin have evidenced that following the phagocytic ability at eliminating cell debris microglia changes to a more inflammatory phenotype (Silva et al., 2010) and even to a senescent-like cell morphology and death if the duration of exposure is prolonged. However, microglia behavior in the presence of unconjugated bilirubin is modulated by the presence of both astrocytes and neurons (Silva et al., 2011). Also to consider is that during the inflammatory phenotype, microglia may intervene in the glutamate homeostasis, but may also contribute to neurite degeneration through the release of NO (Silva et al., 2012). Interestingly, microglia phagocytic features were shown to occur early in ALS disease. Microglia were revealed to aggregate, proliferate, and phagocyte in the lumbar SC of pre-symptomatic mutant SOD1H46R transgenic mice (Sanagi et al., 2010). However, in other studies microglia have shown to contribute to MN death (Dibaj et al., 2011; Brettschneider et al., 2012) and to decrease in number within disease progression (Butovsky et al., 2012), thus contributing to the disease propagation. By using *in vivo* imaging by two-photon laser-scanning microscopy and axonal transection, it was observed different phases of microglia-mediated inflammation in the ALS mice model. Indeed, a first phase (preclinical) with highly reactive microglia was followed by another (clinical stage) with morphologically transformed microglia presenting reduced surveillance activity and reactivity (Dibaj et al., 2011). Regional, temporal, and immune environmental differences may contribute to changes in microglia phenotypes and response heterogeneity, thus requiring differentiated immunomodulatory or even combinatory therapeutic approaches along ALS disease progression.

**FIGURE 2:**
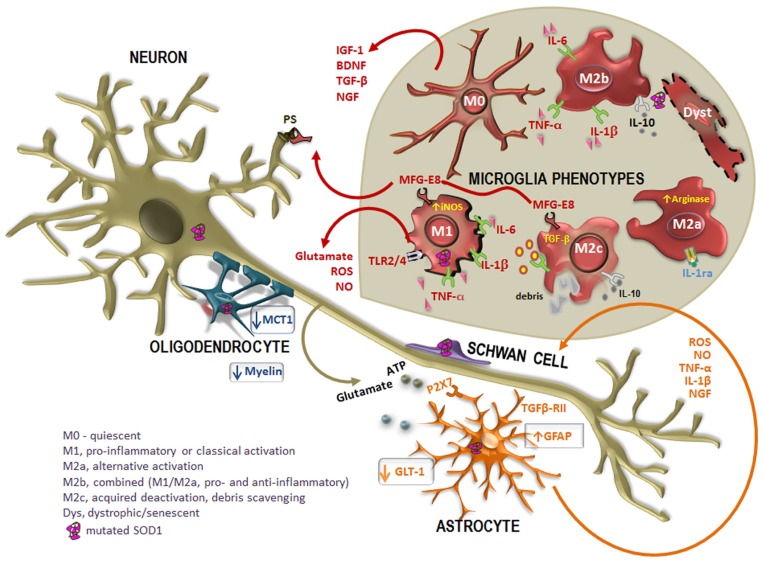
**Altered cross-talk between glial cells and motor neurons (MNs) in ALS disease.** Many studies report the intervention of mutated superoxide dismutase-1 (SOD1)-expressing non-neuronal cells in the pathogenesis of the disease. In case of injury, astrocytes become activated in a process called astrogliosis, characterized by the up-regulation of intermediate filament glial fibrillary acidic protein (GFAP) and increased release of toxic products into the extracellular media, such as pro-inflammatory cytokines [tumor necrosis factor-α (TNF-α), interleukin-1β (IL-1β)] oxidative stressors [reactive oxygen species (ROS), nitric oxide (NO)], as well as glutamate and ATP mediated by the receptor P2X7. In addition, astrocytes also evidence a reduced expression of the glutamate transporter GLT-1, which additionally contribute to neuronal excitotoxicity. However, reactive astrocytes up-regulate nerve growth factor (NGF), which can modulate neuronal survival. Disorganization and destruction of the myelin sheath with a progressive loss of phospholipids and cholesterol is also observed. Moreover, oligodendrocytes also evidence a loss of the of the monocarboxylate transporter 1 (MCT1), thus compromising the energy supply to MNs. On the other hand, increased mutated SOD1 expression in Schwann cells may have intricate ways contributing to slow ALS progression. When mutated SOD1 accumulates within microglia, there is a pattern of activation. Microglia acquire different activation phenotypes (M0, M1, M2a, M2b, M2c, and dystrophic/senescent) and, consequently, produce a diversity of substances that may be either beneficial [insulin-like growth factor 1 (IGF-1), transforming growth factor-β (TGF-β), brain-derived neurotrophic factor (BDNF), and nerve growth factor (NGF)] or toxic (glutamate, ROS, NO) to other cells. Phagocytosis is mediated by the release of milk factor globule-8 (MFG-E8) from microglia M1 and M2c phenotypes that recognizes phosphatidylserine (PS) in apoptotic neurons. Reliable markers for M2a are high IL-1 receptor antagonist (IL-1Ra) and high arginase, for M2b are IL-10, TNF-α, IL-6, and IL-1β, for M2c are TGF-β and IL-10 and for M1 are TNF-α, IL-6, and IL-1β, increased inducible nitric oxide synthase (iNOS) and toll-like receptors (TLR)2 and 4.

***Microglia phenotypes***. Activation of microglia may be observed through the up-regulation of CD11b, Iba1, and CD68 markers. Primary microglia differ from other blood macrophages in the expression of CD11b/CD45^low/high^ and CD68^low/high^ (for review, see Hinze and Stolzing, 2011), although not specific for microglia in a pathologic brain (Matsumoto et al., 2007). These cells show a round morphology with an enlarged cell body and smaller and thicker processes in the resting/quiescent state (M0; **Figure 2**). When activated in response to an insult or injury, microglia are capable of acquiring diverse and complex phenotypes, allowing them to participate in the cytotoxic response, immune regulation, and injury resolution. Microglia may then favor the entrance of inflammatory T cells with which the cells seem to interact. Recent work have classified and characterized M1 and M2 phenotypes (**Table 2**) (for review, see Evans et al., 2013). The first is cytotoxic, characterized by the release of proinflammatory cytokines and influenced by T helper cell type 1 (Th1) that release GM-CSF and interferon-γ (IFN-γ), triggering M1 proliferation. Cytotoxic M1 markers include IL-1β, IL-6, TNF-α, iNOS, COX2, and CX3CL1, and known inducers are the TLR4 agonist LPS and IFN-γ (Chhor et al., 2013).

The M2, promoted by the cytokines IL-4 and IL-13 released by Th2, contribute to neuroprotection once they also secrete anti-inflammatory cytokines, such as IL-4 and IL-10, and growth factors such as insulin-like growth factor 1 (IGF-1). Indeed, IL-4 was shown to protect MNs from the injury produced by LPS-activated microglia (Zhao et al., 2006). Thus, M2 polarization may be desirable, although excessive or prolonged M2 polarization may become prejudicial in allowing unwanted fibrotic responses and scarring, not facilitating axonal growth. However, there are three M2 phenotypes: the M2a or alternate activation repair/regeneration/remodeling phenotype, the M2b immunoregulatory and the M2c acquired-deactivating (**Figure 2**). Reliable markers for M2a stimulated by IL-4 and IL-13 are high IL-1 receptor antagonist (IL-1Ra) and high arginase (Arg1). M2b is stimulated by immune complexes, TLR agonists and IL-1R ligands. Useful markers are IL-1Ra and SOCS3. Characterization of M2c stimulated by IL-10, transforming growth factor-β (TGF-β) and glucocorticoids is obtained through the increased levels of anti-inflammatory cytokines (IL-10, TGF-β), low levels of pro-inflammatory cytokines and enhanced IL-4Rα, Arg1, SOCS3, and CD206 (David and Kroner, 2011; Wilcock, 2012; Chhor et al., 2013). While LPS, IL-1β, TNF-α, and IFN-γ lead to cytotoxic M1 and immunomodulatory M2b activation states, IL-4 mainly triggers M2a phenotype (Chhor et al., 2013).

Therefore, balance between M1 and M2 phenotypes may be a desirable therapeutic goal. Interestingly, mSOD1 microglia isolated from ALS mice at disease onset showed higher levels of Ym1, CD163, and brain-derived neurotrophic factor (BDNF) (M2 markers) and lower levels of Nox2 mRNA (M1 marker) as compared to the end-stage disease (Liao et al., 2012). Interestingly, when co-cultured with wt MNs the first microglia phenotype exerted neuroprotection while the M1 phenotype was neurotoxic, supporting the pathoprogression-related changes in microglia. We may probably consider that microglia display the M2 phenotype at an early stage of the disease switching to the M1 phenotype during the late rapid phase (reviewed in Zhao et al., 2013). However, we should also consider that microglia may become functionally impaired at the end-stage, as recently observed in the SOD1G93A SC microglia (Nikodemova et al., 2013). Different roles of microglia depending on the neurological disease context was recently suggested by Chiu et al. (2013) based on the observation by FACS-transcriptome comparisons that SOD1G93A microglia show a unique phenotype that differs from M1 or M2 macrophages, and from activation with LPS. Robust up-regulation of MMP-12, IGF-1 and osteopontin were pointed as hallmarks. Transcriptomic technology has been carried out to examine the gene expression changes of ALS tissue as compared to controls (reviewed in Heath et al., 2013) and in the future may provide insights into the microglia phenotype profiling prior to disease onset and along ALS progression to assist in the development of new treatments for ALS at different stages.

***Inflammatory microRNA profiling***. MicroRNAs (miRNAs), small, non-coding RNAs, have been recently pointed to mediate cell-to-cell communication (Xu et al., 2013). Inflammatory phenotypes are miR-155, miR-21, miR-146a/b, and miR-124 (Quinn and O’Neill, 2011). Recent studies demonstrated the miR-124 involvement in promoting microglia quiescence by skewing their polarization from an M1 to an M2 phenotype (Willemen et al., 2012) and that miR-155 together with miR-124 are likely to be directly related to M1 and M2 phenotypes, respectively (Cardoso et al., 2012; Ponomarev et al., 2013). Up-regulation of miR-155 is induced by TNF-α and Il-1β (Pottier et al., 2009), as well as by HMGB1 through the activation of the TLR2/MyD88/miR-155 pathway (Wen et al., 2013), while it is down-regulated by TGF-β (Pottier et al., 2009). Increase of miR-146a expression was shown to occur in the aged mice (Rodier and Campisi, 2011), as well as after LPS stimulation (Jiang et al., 2012), and is related with the microglia phagocytic potential (Saba et al., 2012). Moreover, up-regulation of miR-21 in murine models of SC injury was pointed as a modulator of the pro-reactive effects of inflammatory signaling cascades (Nieto-Diaz et al., 2014). Dysregulation of miRNAs expression was lately found in the SC of ALS patients and in microglia isolated from the hSOD1G93A mice. Promising candidates that were found to be altered in patients were miR-146a^*^, miR-524-5p, and miR-582-3p (Campos-Melo et al., 2013), while those up-regulated in the mice model were miR-155, miR-146b, miR-22, miR-365, and miR-125b (Parisi et al., 2013), which need to be further investigated for their relevance as ALS biomarkers and therapeutic targets. Actually, it was recently discovered that inhibition of miR-155 prolongs survival in the mSOD1 mice (Koval et al., 2013).

***Autotaxin***. Autotaxin (ATX) is a secreted lysophospholipase D that converts lysophosphatidylcholine (LPC) to lysophosphatidic acid (LPA), a phospholipid growth factor that activates several transduction pathways, and is involved in migration, proliferation, and survival of various cells (Mori et al., 2011; Furukawa et al., 2013). LPA acts as an autocrine and/or paracrine signaling molecule mediating a broad range of intracellular signaling cascades, especially the RHOA pathway (reviewed in Willier et al., 2011). LPA receptors are expressed by astrocytes, oligodendrocytes, and microglia (Lorenzl et al., 2006; Shi et al., 2010a; He et al., 2013).

Data suggest that ATX levels increase within reactive astrocytes following neurotrauma (Savaskan et al., 2007), but other studies have obtained a down-regulation instead (Shi et al., 2010b). Most fascinating, it was observed that overexpression of ATX inhibits microglial activation and protects the cell against oxidative stress (Awada et al., 2012). Thus, reduced expression of ATX in microglia may contribute to a sustained neuroinflammation condition. Since ATX is also a motility driver that stimulates cell invasion (Sau et al., 2007; Hoelzinger et al., 2008; Magrane and Manfredi, 2009) it can be involved in microglia migration, a property not exploited till now. Although ATX is considered an inflammatory mediator we were not able to find studies demonstrating its relevance or not in ALS.

### NEUROVASCULAR CHANGES

BBB and BSCB are dynamic and complex interfaces between the blood and the CNS. Endothelial cells and tight junctional complexes physically limit solute exchanges between the blood and the brain. These cells together with pericytes, astrocytes, neurons, microglia, and oligodendrocytes and the basement membrane form the neurovascular unit (for review, see Cardoso et al., 2010; Sá-Pereira et al., 2012). The interaction between all these components provides a sustainable environment for neural function while restricting permeability and transport. Alterations in barrier properties and dynamics were observed in transgenic SOD1 rats (Andjus et al., 2009; Nicaise et al., 2009). In particular, it was noticed a reduction of endothelial tight junctions (Zhong et al., 2008), together with the disruption of the neurovascular unit and up-regulation of MMP-9 (Miyazaki et al., 2011) prior to MN degeneration. MMP-9 activation was observed in blood vessel-like structures and microglia. Increased microvascular microglia, expressing CX3CR1 and weakly labeling Iba1, were detected in the SCs from the ALS mice model and suggested to have a bone marrow origin (Lewis et al., 2009). In addition, it was recently demonstrated that SC microglia in the mSOD1 mice promote the recruitment of inflammatory monocytes into the CNS well before the onset of the disease, triggering microglia apoptosis (**Table 2**) (Butovsky et al., 2012). Actually, the BSCB has been shown to be disrupted in different mSOD1 mice before MN degeneration, thus favoring blood monocytes infiltration (Barbeito et al., 2010). To that it may account the 54% reduction in pericytes at the BSCB level in patients with ALS (Winkler et al., 2013). In fact, some authors consider ALS as a neurovascular disease (Garbuzova-Davis et al., 2012) with evidence indicating impairment of all neurovascular unit components, including the BBB and the BSCB in both ALS patients and animal models. Disruption of BBB and BSCB was observed in the G93A mice by electron microscopy in places of MN degeneration at both early and late disease stages (Garbuzova-Davis et al., 2007). The damage of barriers besides allowing the entrance of T lymphocytes, such as CD4^+^ (helper/inducer) and CD8^+^ (cytotoxic) into the brain parenchyma, may also permit the entrance of harmful substances that will contribute to disrupt neuronal homeostasis and accelerate MN degeneration. The impact of microvascular damage in ALS pathology is a novel and promising research topic in that interactions with glia may determine neuroprotective or cytotoxic cell phenotypes with consequences on MN survival.

## INFLAMMATORY COMPONENTS IN ALS AND PATHOLOGICAL CELL–CELL COMMUNICATION

Communication between neurons and microglia is essential for maintaining homeostasis in the CNS and altered cross-talk is implicated in the pathogenesis of ALS and other MN diseases (Appel et al., 2011). Dysregulated neuron–neuron signaling and neuron–microglia cross-talk in ALS, and other neurodegenerative diseases, may derive from: (i) secretion of toxic mediators or fail to secrete trophic factors, or both, resulting in reduced function and survival of MNs; (ii) SFs released by neurons and their action on microglia receptors; (iii) changes in direct cellular interactions.

Reduced expression of trophic factors such as BDNF, fibroblast growth factor 2 (FGF2), and IGF-1 was found in the SC of the newborn rat after intrathecal administration of CSF from ALS patients (Deepa et al., 2011). To note, that the release of mSOD1 was shown to trigger microgliosis and neuronal death (Urushitani et al., 2006) and increased levels of ROS/RNS, mainly of NO, were shown to play a critical role in the earliest stages of neuronal dysfunction in ALS (Drechsel et al., 2012). NO mainly produced by glia through the activation of iNOS can react with superoxide producing the potent oxidant peroxynitrite (Beckman et al., 1990), which mediates apoptosis or necrosis, depending on the concentration (Bonfoco et al., 1995). Peroxynitrite also induces toxic glial phenotypes further propagating oxidative damage and cellular dysfunction. Interestingly, the same concentrations of NO may either promote the survival of MNs healthy conditions or induce apoptosis and glia reactivity when in the presence of stressors. Up-regulation of neuronal NO synthase (nNOS) was shown to occur in MNs before iNOS in either pre-symptomatic ALS animal models or in patients and to be associated with MN loss (Moreno-Lopez et al., 2011; Drechsel et al., 2012).

Activated microglia and astrocytes amplify the initial damage to the MNs by activating AP-1 and NF-κB through production of proinflammatory cytokines and apoptosis-triggering molecules such as TNF-α and Fas ligand (FASL). TNF-α and IL-1β exert neurotoxic effects *in vitro*, but deletion of the individual genes seems to not affect the course of the disease. In addition, dying MNs release ATP that can further activate glia through the purinergic receptor P2X7 expressed by both microglia and astrocytes (**Figure 2**) (Gandelman et al., 2010; Glass et al., 2010). Classical microglia activation results in upregulation of MHC class II proteins that are involved in presentation of antigens to T lymphocytes. Microglia activation is mediated by the release of SFs and/or expression of surface receptors by neurons and astrocytes, such as CX3CL1, CD200, and CCL21. Microglia also express a diverse set of pattern recognition receptors (PRRs) for pathogen-associated molecular patterns (PAMPs) and endogenous ligands derived from injury that include TLRs and inflammasome. In addition, emerging evidence suggests that members of the nuclear receptor (NR) family of transcription factors, many of which are ligand-dependent, control the activation of microglia under physiological and pathological conditions (for review, see Saijo et al., 2013).

Neuroinflammation involves the activation and proliferation of microglia and the infiltration of T cells into the brain and SC. In these conditions, astrocytes and microglia release IL-1β, TNF-α, and IL-6 (**Figure 2**). Cytokines can then up-regulate oxidative stress by NO and superoxide (O_2_-) generation (for review, see Philips and Robberecht, 2011). Repair and limitation of the damage is promoted by the release of trophic and anti-inflammatory factors. Deleterious microglia M1 and benign M2 phenotypes are influenced by astrocytes and T cell subsets. In ALS, it is still currently unknown the precise function of microglia and astrocytes and how they mediate neuroinflammation and contribute to the pathology. Identification of the factors driving microglia phenotype and consequent functional changes, once known, may advance our knowledge on their role and on ways to modulate these cells. In addition, it will be important to clarify whether inflammation contributes to ALS pathogenesis or in opposite is a protective response, and if different individual immune responses to the disease are also implicated, so that immunomodulatory therapies can be pursued.

### MICROGLIA–T CELL CROSS-TALK

Evidence for autoimmunity in ALS was proposed in 1990s (for review, see Appel et al., 1993). Inflammation is not a resultant from MN degeneration since it regulates the balance between neuroprotection and neurotoxicity (Henkel et al., 2009). Initially, the diverse populations and phenotypes of CD4^+^ T cells that cross-talk with microglia can slow disease progression, but later they may contribute to the acceleration of the disease. The inflammatory process involves infiltration of T cell subpopulations at sites of neuronal injury in the brain parenchyma. T cells may damage MNs by cell–cell contact or cytokine secretion through the activation of microglia (Holmoy, 2008). CD8^+^ cytotoxic T cells and natural killer T (NKT) cells were found significantly increased in patients with ALS, while regulatory T (Treg) cells were decreased (Rentzos et al., 2012). It was suggested that CD4^+^ T cells may trigger oxidative phosphorylation in microglia and CD8^+^ may stimulate phagocytosis accordingly to data obtained by FACS-transcriptome comparisons in cells isolated from the SC of SOD1G93A mice (Chiu et al., 2013).

The naive T cells, or Th0 cells, expand and differentiate into at least four functionally distinct subsets upon stimulation: Th1, Th2, Treg, and Th17 cells. Th1 cells secret IFN-γ and turn resting microglia into M1 phenotype; in contrast Th2 and Treg cells release IL-4 which induces activation of resting microglia into M2 phenotype (for review, see Lewis et al., 2012). Th1 and Th17 are CD4^+^ T cells that produce proinflammatory cytokines, causing damage while CD4^+^CD25^h^^igh^ T lymphocytes (Treg) suppress Th1 cell effector function (Dittel, 2008).

Interestingly, it was demonstrated that mSOD1 mice lacking CD4^+^ T cells evidence a faster disease progression and decreased microglia reactivity and as so, future therapeutic interventions should consider the benefits that T cells may have (Beers et al., 2008; Chiu et al., 2008; Zhao et al., 2013). Compromise of Treg lymphocytes with disease progression diminishes the secretion of IL-4 and fails in suppressing the toxic properties of microglia (Beers et al., 2011a). Indeed, mSOD1 Treg suppress immune toxicity by inhibiting microglial activation, CD4^+^CD25^-^ (T effector cells) proliferation, and the accompanying cytotoxicity, thus providing MN protection in ALS (Zhao et al., 2012). As disease progresses, the supportive Treg/Th2/M2 changes to an injurious Th1/M1 response triggering increased TNF-α secretion that was shown to induce the dysfunction of Tregs (Zhao et al., 2013). Therefore, elevation of Tregs in patients with ALS may trigger a longer life expectancy.

### MN–ASTROCYTE CROSS-TALK

It was before considered that the mutant protein was the responsible for the disease onset in MNs with microglia and astrocytes only determining disease progression and extent. However, expression of mSOD1 in microglia and astrocytes is now being related with the disease onset and early stage disease, while healthy SOD1 in those cells was shown to delay ALS progression.

Several studies evidenced the demise of MNs in the presence of astrocytes harboring SOD1 mutations (Yamanaka et al., 2008b), attesting the non-cell-autonomous pathology in ALS, i.e., degeneration of MNs requires mSOD1 expression in other cells additionally to neurons. This finding was similarly obtained for astrocytes derived from ALS patients, which have shown to cause MN death (Haidet-Phillips et al., 2011). Deregulation between astrocytes and MNs communication seems to involve TGF-β signaling pathways (Phatnani et al., 2013) and miR-124a mediated regulation (Morel et al., 2013). Evidence for the role of miRNAs in MN diseases is substantiated by the relevance that the proteins TDP-43 and FUS/TLS, responsible for the processing of miRNAs, RNA maturation and splicing (Kiernan et al., 2011), have recently acquired in ALS (Lagier-Tourenne and Cleveland, 2009; Haramati et al., 2010). A major challenge would be to establish circulating miRNAs as particularly accessible biomarkers to monitor ALS.

Activated astrocytes (see Astrocyte Reactivity for more details) may also interfere with MNs function due to the reduced secretion of trophic factors such as BDNF, glial cell line-derived neurotrophic factor (GDNF) and vascular endothelial growth factor (VEGF), but this is not a clarified issue yet (for review, see Evans et al., 2013).

### REGULATION OF ASTROCYTE–MICROGLIA INTERCONNECTIVITY ACTIVATION

Both *in vitro* and *in vivo* experiments have shown that astrocytes and microglia containing mSOD1 exert deleterious effects on MNs, by releasing proinflammatory factors (Phani et al., 2012). Microglia may respond earlier than astrocytes to injury and stress by first activating NF-κB and mitogen-activated protein kinase (MAPK) signaling pathways, thus leading to a faster release of TNF-α and IL-1β (Brites, 2012). In that way one may believe that the activation of microglia precedes the reactivity of astrocytes and depends from the factors and cytokines that microglia release. Curiously, TNF-α and IL-1β released from activated microglia were reported to produce an inhibitory effect on Cx-43 expression, the main constitutive protein of gap junctions. Therefore, blockage of communication between astrocytes by the activated microglia can contribute to decrease the neuroprotective role of astrocytes (Meme et al., 2006). When considering the p25-mediated neuroinflammation, astrogliosis was shown to precede microglia activation, and apparently mediate the production of LPC, which as a chemoattractant for T cells may recruit peripheral cells into the brain (Sundaram et al., 2012). Recently, it was shown that astrocytes depend on functional microglia for response to LPS and to TLR2, 3, and 4 ligation. In their absence, astrocytes did not respond to TLR4 ligation and only weakly responded to TLR2 and 3 ligation (Holm et al., 2012). Thus, activation of astrocytes may be modulated by the proinflammatory cytokines released from microglia in an attempt to diminish the extent of excitotoxicity (Tilleux et al., 2007), but they can modulate microglia activation, as well (Welser and Milner, 2012). Which cells between microglia and astrocytes are first activated by the aggregated mSOD1 is a very controversial issue since different studies have obtained diverse profiles of glial activation in ALS models along disease progression. Some studies point out that microglia activation precede astrocyte reactivity (Alexianu et al., 2001). Others that astrogliosis is initiated in the symptomatic phase, while prominent microgliosis is only evident later at the moribund phase (Yang et al., 2011). However, since a close interaction/communication of both cells occurs in ALS, a better understanding of the benefits and risks of astrocyte and microglia activation in ALS will help to determine whether therapeutic strategies should envisage enhancing or impairing the actions of glial cells in ALS.

### MN–MICROGLIA SIGNALING

Communication between MNs and microglia is essential for maintaining local homeostasis during both physiological and inflammatory conditions. Neuroprotective signaling from MN to microglia involve FKN and CD200, as previously mentioned. In addition, microglia “calming” effects during neuroinflammation are mediated through ATX (Awada et al., 2012). Insights into the complex intercellular perturbations and influence of alarming and calming factors underlying neurodegeneration will enhance our efforts toward target-driven directed therapeutic strategies in ALS.

#### CX3CR1 deficiency

The neuroprotective/neurotoxic role of CX3CL1/CX3CR1 signaling is still a matter of debate once it seems to depend upon the CNS insult (for review, see Desforges et al., 2012). Deletion of CX3CR1 in a transgenic model of ALS mice was shown to extend neuronal cell loss, suggesting that CX3CL1/CX3CR1 signaling limits microglial toxicity in ALS (Cardona et al., 2006). Interestingly, it was also shown that treatment with LPS down-regulated the expression of CX3CR1, thus suppressing the functional response to FKN (Boddeke et al., 1999) and potentiating LPS neurotoxic effects (Zujovic et al., 2000). Accordingly, in ALS, the disruption of CX3CL1/CX3CR1 signaling evidenced to promote neurodegeneration following LPS administration (Cardona et al., 2006).

#### Cathepsin S influence

Although it has been proposed that cathepsins compensate for each other because of their overlapping substrate specificities, there is increasing evidence that disturbance of the normal balance and extralysosomal localization of cathepsins contribute to age-related diseases (for review, see Nakanishi, 2003). CatS that is a lysosomal/endosomal cysteine protease that degrades extracellular matrix proteins, even at neutral pH, was shown to be increasingly expressed by microglia upon LPS surcharge (Petanceska et al., 1996). However, some authors indicate that a further challenge with ATP is required to observe CatS release (Clark and Malcangio, 2012). This protease is preferentially expressed by antigen-presenting cells, including microglia.

Microglial CatS seems to be responsible for the liberation of neuronal FKN (**Figure 1**), which via the specific receptor CX3CR1 in microglia activate p38 MAPK pathway leading to the release of mediators that interact with neurons (Clark et al., 2007). Several inhibitors have been tried to regulate the immune modulatory effects of CatS (Clark and Malcangio, 2012) and a shift from a Th1/Th17 type response to a Th2 type of response was obtained (Baugh et al., 2011).

The up-regulation of cathepsins appears as a general transcriptional event in ALS (Gonzalez de Aguilar et al., 2008; Offen et al., 2009; Boutahar et al., 2011). However, reference to CatS and microglia activation was not until now indicated in the context of ALS. Therefore, if CX3CL1/CX3CR1 demonstrates to have a role in any stage of ALS progression, inhibition of CatS may constitute a therapeutic approach for ALS.

#### Exosomes and disease spread

Exosomes are secretory vesicles deriving from late endosomes and multivesicular bodies that mediate neuron–glia communication, have 50–100 nm in size and carry specific protein and RNA cargo (Fitzner et al., 2011; Fruhbeis et al., 2012; El Andaloussi et al., 2013). Exosomes contain both miRNAs and mRNAs that may be delivered and be functional in another cell (Valadi et al., 2007). Selectivity for miRNA incorporation into exosomes is proposed based on the fact that some are exclusive in exosomes derived from immature dendritic cells while others only exist in those from mature dendritic cells (Stoorvogel, 2012). Indeed, exosomes and miRNAs have been found to participate in cellular senescence and contribute to aging (Xu and Tahara, 2013). In addition senescent cells produce high levels of exosomes thus interacting and inducing the senescence of neighboring cells. As so, exosomal miRNAs might become useful biomarkers of disease. Recently, it was suggested that exosomes may be important candidates to deliver siRNA (El Andaloussi et al., 2013) and specific drugs, based on the perceived advantages of nanoparticle size and non-cytotoxicity (Sun et al., 2013), thus constituting a therapeutic platform.

Microglia was shown to internalize oligodendroglial exosomes, thus participating in the degradation of oligodendroglial membrane (Fitzner et al., 2011). However, microglia also release exosomes (Potolicchio et al., 2005; Hooper et al., 2012), which generate IL-1β to the extracellular environment propagating inflammation (Turola et al., 2012). Increased exosome discharge by microglia was observed after stimulation with α-synuclein and such activated exosomes revealed an increased membrane content in TNF-α (Chang et al., 2013). Interestingly, it was shown that mouse MN-like NSC-34 cells overexpressing hSOD1G93A secrete SOD1 via exosomes probably accounting to cell–cell-mediated mutant toxicity in ALS pathogenesis (Gomes et al., 2007). Therefore, exosomes from microglia may spread pathogenic factors, such as SOD1 and promote inflammation while influencing neuronal survival (Chang et al., 2013). In line with this, a latest study reports that cell-to-cell transmission of SOD1 misfolding is mediated by two non-exclusive mechanisms: through the release of protein aggregates that are taken up by macropinocytosis or via exosomes secreted from living cells (Grad et al., 2014).

## CHALLENGES TO NERVE REGENERATION IN ALS

Reconstruction of neural network implies the restoration of tissue architecture and cell functionality. Cell replacement-based repair strategies have been tested both *in vitro* and *in vivo* and a major challenge resides in maintaining the cell function when transplanted to a broken parenchyma homeostasis. Confront with excessive neuroinflammation and cell senescence may compromise the success of the strategy. In particular, reactive astrocytes exacerbate inflammation and by forming glial scars impede regenerating axons from traversing the lesions, while myelin debris prevent axon growth and microglia lose the ability to migrate, phagocyte or sustain inflammation from spread. Therefore, the rewiring of the CNS to foster a permissive environment for neuroregeneration is the key to a successful functional integration and repair (Xu et al., 2011; Kim et al., 2012).

### CELL SENESCENCE

Redox changes within neurons (Olivieri et al., 2001), ER stress condition (Di Virgilio, 2000), and mitochondria dysfunction (Konnecke and Bechmann, 2013) are accelerated by aging and are emerging as common features relevant to the pathogenesis of neurological disorders, including ALS. Actually, the variation in SOD1 activity in aging ALS patients, when compared to younger ones, point to an increased oxidative misbalance vulnerability (Fiszman et al., 1999). Nevertheless, it was recently indicated that the ALS incidence decline in the elderly (Schoser and Blottner, 1999; Demestre et al., 2005), suggesting that the disease is not merely the result of aging. However, markers of senescence were found increased in satellite cells from ALS muscle biopsies suggesting a vulnerability to muscle atrophy (Gottschall and Deb, 1996). In addition, autophagic dysfunction and mitochondrial DNA damage in the CNS are prominently found in microglia with aging and may lead to a defective turnover of mitochondria and accumulation of hypergenerated ROS (reviewed in Nakanishi and Wu, 2009). Interestingly, dysfunctional and senescent microglia may even release compounds that inhibit neuronal autophagy (Alirezaei et al., 2008) and neurogenesis (reviewed in Wong, 2013). Given the important and necessary functions of microglia in the CNS homeostasis it is of major relevance to understand the multiple stage-related microglia phenotypes in ALS, including the increased vulnerability to a senescent cell with the disease progression, as observed in other neurodegenerative and age-related CNS disorders (Luo et al., 2010; Kaplan et al., 2014).

#### Microglia degeneration

Recent data obtained in the mSOD1 mouse model suggest a dominant neuroinflammatory response in the CNS, with a reactive microglia in preclinical stages that turns into an irresponsive cell during disease progression, and a degenerative process in the PNS (Dibaj et al., 2011). Earlier studies evidenced that mutated SOD1 microglia have an age-dependent cytotoxic potential which reveals upon a stimulatory effect (Weydt et al., 2004). Interestingly, extracellular SOD1G93A mediates the activation of CD14–TLR2 pathway with the consequent release of TNF-α and IL-1β, thus propagating the proinflammatory stimuli (Bowerman et al., 2013). However, since immunosuppressive strategies have not proven consistent efficacy (Appel et al., 1993, 2011; Baugh et al., 2011), one may believe that microglia function may change along disease progression with consequent differential effects on MNs. Late data evidenced that SC microglia proliferate and that TNF-α mRNA expression decreases during disease progression in SOD1G93A rats (Nikodemova et al., 2013). Indeed, microglia revealed to not be polarized to M1 or M2 phenotypes at any disease stage and CNS region evaluated. In addition, SC microglia evidenced to be irresponsive to experimental systemic inflammation at ALS end-stage. Diminished glial neuroprotection by senescent and/or dysfunctional microglia has been suggested to play a role in neurodegenerative diseases, mainly in the late stage (Streit, 2006). In such circumstances the aged microglia evidenced a “dystrophic” morphology with the loss of finely branched cytoplasmic processes, cytoplasmic beading/spheroid formation, and cytoplasmic fragmentation (cytorrhexis; **Figure 2**) (Streit et al., 2004). Such severe abnormalities in microglia, including cell fusion (multinucleated giant cells) at the symptomatic stage and cytorrhexis at the end stage are indicative of microglial aberrant activation and degeneration, respectively, and were observed in SOD1G93A transgenic rats (Fendrick et al., 2007). Although prevalent in older human subjects this dystrophic cells may also in rare instances be observed in the young brain (Luo and Chen, 2012). Pathogenic miRNAs, such as miR-155, miR-146a, and miR-124 in microglia may be associated with the acquisition of a senescent phenotype (Saba et al., 2012; Ponomarev et al., 2013). We recently observed that aged-cultured microglia exhibit lower phagocytic ability and higher expression levels of miR-146a than younger cells (Caldeira et al., 2013). Whether these findings are related with a dysfunctional microglia at ALS end-stages deserve to be further investigated.

#### Demyelination progression

Although new oligodendrocytes were observed in the SC of SOD1G93A mice, they do not fully maturate, resulting in progressive demyelination and accelerated disease (Liu et al., 2002). The authors also found similar myelination defects in postmortem samples taken from SC and motor cortex from ALS patients. Actually, there are many potent inhibitors of axonal regeneration in the injured CNS including myelin-associated proteins, fibrinogen, and axonal guidance molecules, where epidermal growth factor receptor (EGFR) and eukaryotic ribosome biogenesis protein 1 (Erb1) may have a special role in reducing the effects of multiple inhibitors of axonal regeneration (Arthur-Farraj et al., 2012; Leinster et al., 2013). However, the role of EGFR on the protection of MN synapses and survival extension in SOD1G93A mice is still a matter of debate; hence, it was reported that EGFR inhibitors failed to extend ALS mouse survival although influencing disease progression (Le Pichon et al., 2013).

Another important point to be considered is the decrease in the phagocytic clearance by the dysfunctional microglia that results in the accumulation of myelin debris, leading to oligodendrocyte differentiation arrest and decreased recruitment of oligodendrocyte precursor cells (Walter and Neumann, 2009). Activated microglia were also shown to attenuate the proliferation of the oligodendrocyte precursor cells, thus concurring for demyelination progression (Taylor et al., 2010), and reinforcing the recovery of healthy microglia as a potential therapeutic target in ALS. Indeed, studies on the global gene expression during demyelination and remyelination by microarray analysis reinforced that the primary function of microglia is the tolerance induction and support to regeneration (Olah et al., 2012).

### CELL REPLACEMENT THERAPY

Cell replacement therapy has been suggested as a promising strategy for MN disease. The use of combined strategies to restore both the healthy state of MNs and glial cells, such as microglia, and their correct cross-talk to face persistent neurotoxic insults may even provide better benefits for this devastating disease.

Mesenchymal stem cells (MSCs) isolated from the bone marrow of ALS patients did not show morphological or functional differences from those obtained from donors and seem to be useful for cell-based therapy for ALS patients (Hadass et al., 2013). In a few well-monitored ALS patients the autologous transplantation of such MSCs into the SC evidenced to be well tolerated and to promote some clinical improvement (Min et al., 2012).

Another proposed strategy is the olfactory ensheathing cell (OEC) transplantation that although evidencing to slow the rate of ALS progression in a short period (Inoue et al., 2003) and to improve pulmonary function (Guegan et al., 2001) has been a matter of debate. Indeed, prominent glial and inflammatory reaction around the brain delivery track was observed in postmortem samples (Reyes et al., 2010).

Efficacy of transplantation-based astrocyte replacement was also evidenced as a promising therapy for slowing focal MN loss associated with ALS while also reducing microgliosis in the hSOD1G93A rodents (Lepore et al., 2008). Effects may derive from the release of growth factors that are decreased in ALS and already evidenced to increase mSOD1 mice survival (Kaspar et al., 2003; Park et al., 2009). However, limited efficacy was obtained in a later study (Lepore et al., 2011). The use of induced pluripotent stem cell (iPSC) technologies may allow in the future the autologous cell transplantation in ALS patients, including MNs (Papadeas and Maragakis, 2009).

More recently, depletion of microglia cells expressing mSOD1 with clodronate liposomes and subsequent transplantation with bone marrow cells (BMCs) expressing wtSOD1 was shown to trigger microglia replacement and to slow ALS disease progression in the SOD1G93A mice model (Lee et al., 2012). The method seems to afford better therapeutic effects than the one by Ohnishi et al. (2009) using BMCs transplantation since microglia renewal is better achieved by tissue-resident microglia rather than by BMCs (Davoust et al., 2008). Overall, the mechanisms and functional implications of microglia replacement require further elucidation, inasmuch because myeloid-derived infiltrating cells (monocyte-derived macrophages) revealed to be functionally distinct from the resident microglia assisting them (Jung and Schwartz, 2012; London et al., 2013). Clearly, additional research is required to address these issues and contribute to develop strategies able to stop, or at least delay, ALS progression.

## CONCLUSION

Amyotrophic lateral sclerosis is a fatal neurodegenerative disorder with limited identified targets, biomarkers, and therapeutic options. Therefore, a better comprehension of the underlying molecular mechanisms is necessary to develop novel etiological therapeutic strategies. Multiple studies suggest that a complex pathological interplay between MNs and glial cells, involving neuroinflammation and microglia physiopathological changes drive the performance of these glial cells before the ALS onset and during disease progression till late and end stages. In this context, we here summarized the growing body of evidence supporting the key role of microglia in the deregulated motor-neuron interconnectivity and in the dreadful chain of events leading to MN degeneration in ALS. As such, insights into the complex intercellular perturbations underlying ALS disease and centered on microglia phenotypic changes, and associated detrimental functions, will help on our efforts to develop effective therapeutic approaches for recovering adequate microglia function in initiation and progression phases of ALS. In conclusion, the role of microglia in keeping brain homeostasis leads to consider that healthy microglia may be used to replace the senescent or irresponsive cell. In addition, dysfunctional microglia may be differently modulated, either directly or indirectly, to be transformed in a less reactive cell to challenges in excessive and chronic neuroinflammation, or alternatively rejuvenated to enhance their capacity to fight the insult and improve disease outcomes. Considerably more research is necessary to realize the feasibility and usefulness of such strategies before their potential in clinic can be realized.

## Conflict of Interest Statement

The authors declare that the research was conducted in the absence of any commercial or financial relationships that could be construed as a potential conflict of interest.

## References

[B1] Alexianu M. E. Kozovska M. Appel S. H. (2001). Immune reactivity in a mouse model of familial ALS correlates with disease progression. *Neurology* 57 1282–1289 10.1212/WNL.57.7.1282 11591849

[B2] Alirezaei M. Kiosses W. B. Flynn C. T. Brady N. R. Fox H. S. (2008). Disruption of neuronal autophagy by infected microglia results in neurodegeneration. *PLoS ONE* 3:e2906 10.1371/journal.pone.0002906 PMC2483417 18682838

[B3] Andjus P. R. Bataveljic D. Vanhoutte G. Mitrecic D. Pizzolante F. Djogo N. (2009). In vivo morphological changes in animal models of amyotrophic lateral sclerosis and Alzheimer’s-like disease: MRI approach. *Anat. Rec. (Hoboken)* 292 1882–1892 10.1002/ar.20995 19943341

[B4] Andrus P. K. Fleck T. J. Gurney M. E. Hall E. D. (1998). Protein oxidative damage in a transgenic mouse model of familial amyotrophic lateral sclerosis. *J. Neurochem.* 71 2041–2048 10.1046/j.1471-4159.1998.71052041.x 9798929

[B5] Appel S. H. Smith R. G. Engelhardt J. I. Stefani E. (1993). Evidence for autoimmunity in amyotrophic lateral sclerosis. *J. Neurol. Sci.* 118 169–174 10.1016/0022-510X(93)90106-9 8229065

[B6] Appel S. H. Zhao W. Beers D. R. Henkel J. S. (2011). The microglial–motoneuron dialogue in ALS. *Acta Myol.* 30 4–8 21842586 PMC3185827

[B7] Arthur-Farraj P. J. Latouche M. Wilton D. K. Quintes S. Chabrol E. Banerjee A. (2012). c-Jun reprograms Schwann cells of injured nerves to generate a repair cell essential for regeneration. *Neuron* 75 633–647 10.1016/j.neuron.2012.06.021 22920255 PMC3657176

[B8] Awada R. Rondeau P. Gres S. Saulnier-Blache J. S. Lefebvre D’Hellencourt C. Bourdon E. (2012). Autotaxin protects microglial cells against oxidative stress. *Free Radic. Biol. Med.* 52 516–526 10.1016/j.freeradbiomed.2011.11.014 22155714

[B9] Barbeito A. G. Mesci P. Boillee S. (2010). Motor neuron-immune interactions: the vicious circle of ALS. *J. Neural. Transm.* 117 981–1000 10.1007/s00702-010-0429-0 20552235 PMC3511247

[B10] Barbosa M. I. (2013). *Dissecting Cross-talk Between Microglia and Motoneurons in ALS: Signalling Events and Soluble Factors*. Master’s thesis, Faculdade de Ciências e Tecnologia, Universidade Nova de Lisboa, Lisbon

[B11] Baugh M. Black D. Westwood P. Kinghorn E. Mcgregor K. Bruin J. (2011). Therapeutic dosing of an orally active, selective cathepsin S inhibitor suppresses disease in models of autoimmunity. *J. Autoimmun.* 36 201–209 10.1016/j.jaut.2011.01.003 21439785

[B12] Bazan J. F. Bacon K. B. Hardiman G. Wang W. Soo K. Rossi D. (1997). A new class of membrane-bound chemokine with a CX3C motif. *Nature* 385 640–644 10.1038/385640a0 9024663

[B13] Beckman J. S. Beckman T. W. Chen J. Marshall P. A. Freeman B. A. (1990). Apparent hydroxyl radical production by peroxynitrite: implications for endothelial injury from nitric oxide and superoxide. *Proc. Natl. Acad. Sci. U.S.A.* 87 1620–1624 10.1073/pnas.87.4.1620 2154753 PMC53527

[B14] Beers D. R. Henkel J. S. Xiao Q. Zhao W. Wang J. Yen A. A. (2006). Wild-type microglia extend survival in PU.1 knockout mice with familial amyotrophic lateral sclerosis. *Proc. Natl. Acad. Sci. U.S.A.* 103 16021–16026 10.1073/pnas.0607423103 17043238 PMC1613228

[B15] Beers D. R. Henkel J. S. Zhao W. Wang J. Appel S. H. (2008). CD4^+^ T cells support glial neuroprotection, slow disease progression, and modify glial morphology in an animal model of inherited ALS. *Proc. Natl. Acad. Sci. U.S.A.* 105 15558–15563 10.1073/pnas.0807419105 18809917 PMC2547419

[B16] Beers D. R. Henkel J. S. Zhao W. Wang J. Huang A. Wen S. (2011a). Endogenous regulatory T lymphocytes ameliorate amyotrophic lateral sclerosis in mice and correlate with disease progression in patients with amyotrophic lateral sclerosis. *Brain* 134 1293–1314 10.1093/brain/awr074 21596768 PMC3097891

[B17] Beers D. R. Zhao W. Liao B. Kano O. Wang J. Huang A. (2011b). Neuroinflammation modulates distinct regional and temporal clinical responses in ALS mice. *Brain Behav. Immun.* 25 1025–1035 10.1016/j.bbi.2010.12.008 21176785 PMC3096756

[B18] Benkler C. Ben-Zur T. Barhum Y. Offen D. (2013). Altered astrocytic response to activation in SOD1(G93A) mice and its implications on amyotrophic lateral sclerosis pathogenesis. *Glia* 61 312–326 10.1002/glia.22428 23280929

[B19] Bertolin C. D’Aascenzo C. Querin G. Gaiani A. Boaretto F. Salvoro C. (2013). Improving the knowledge of amyotrophic lateral sclerosis genetics: novel SOD1 and FUS variants. *Neurobiol. Aging* 35 1212.e7–1212.e10 10.1016/j.neurobiolaging.2013.10.093 24325798

[B20] Bianchi M. E. (2009). HMGB1 loves company. *J. Leukoc. Biol.* 86 573–576 10.1189/jlb.1008585 19414536

[B21] Bianchi M. E. Manfredi A. A. (2009). Immunology. Dangers in and out. *Science* 323 1683–1684 10.1126/science.1172794 19325105

[B22] Boddeke E. W. Meigel I. Frentzel S. Biber K. Renn L. Q. Gebicke-Harter P. (1999). Functional expression of the fractalkine (CX3C) receptor and its regulation by lipopolysaccharide in rat microglia. *Eur. J. Pharmacol.* 374 309–313 10.1016/S0014-2999(99)00307-6 10422773

[B23] Boillée S. Vande Velde C. Cleveland D. W. (2006a). ALS: a disease of motor neurons and their nonneuronal neighbors. *Neuron* 52 39–59 10.1016/j.neuron.2006.09.018 17015226

[B24] Boillée S. Yamanaka K. Lobsiger C. S. Copeland N. G. Jenkins N. A. Kassiotis G. (2006b). Onset and progression in inherited ALS determined by motor neurons and microglia. *Science* 312 1389–1392 10.1126/science.1123511 16741123

[B25] Bonfoco E. Krainc D. Ankarcrona M. Nicotera P. Lipton S. A. (1995). Apoptosis and necrosis: two distinct events induced, respectively, by mild and intense insults with *N*-methyl-D-aspartate or nitric oxide/superoxide in cortical cell cultures. *Proc. Natl. Acad. Sci. U.S.A.* 92 7162–7166 10.1073/pnas.92.16.7162 7638161 PMC41299

[B26] Bourd-Boittin K. Basset L. Bonnier D. L’helgoualc’h A. Samson M. Théret N. (2009). CX3CL1/fractalkine shedding by human hepatic stellate cells: contribution to chronic inflammation in the liver. *J. Cell. Mol. Med.* 13 1526–1535 10.1111/j.1582-4934.2009.00787.x 19432809 PMC3828864

[B27] Boutahar N. Wierinckx A. Camdessanche J. P. Antoine J. C. Reynaud E. Lassabliere F. (2011). Differential effect of oxidative or excitotoxic stress on the transcriptional profile of amyotrophic lateral sclerosis-linked mutant SOD1 cultured neurons. *J. Neurosci. Res.* 89 1439–1450 10.1002/jnr.22672 21647936

[B28] Bowerman M. Vincent T. Scamps F. Perrin F. E. Camu W. Raoul C. (2013). Neuroimmunity dynamics and the development of therapeutic strategies for amyotrophic lateral sclerosis. *Front. Cell. Neurosci.* 7:214 10.3389/fncel.2013.00214 PMC3833095 24312006

[B29] Brettschneider J. Toledo J. B. Van Deerlin V. M. Elman L. Mccluskey L. Lee V. M. (2012). Microglial activation correlates with disease progression and upper motor neuron clinical symptoms in amyotrophic lateral sclerosis. *PLoS ONE* 7:e39216 10.1371/journal.pone.0039216 PMC3375234 22720079

[B30] Brites D. (2012). The evolving landscape of neurotoxicity by unconjugated bilirubin: role of glial cells and inflammation. *Front. Pharmacol.* 3:88 10.3389/fphar.2012.00088 PMC3361682 22661946

[B31] Butovsky O. Siddiqui S. Gabriely G. Lanser A. J. Dake B. Murugaiyan G. (2012). Modulating inflammatory monocytes with a unique microRNA gene signature ameliorates murine ALS. *J. Clin. Invest.* 122 3063–3087 10.1172/JCI62636 22863620 PMC3428086

[B32] Caldeira C. Frederico A. Vaz A. Fernandes A. Brites D. (2013). Age-related differences in microglia reactivity: relevance to Alzheimer’s disease. *J. Neurochem.* 125 (Suppl. 1) 185

[B33] Calvo M. Zhu N. Tsantoulas C. Ma Z. Grist J. Loeb J. A. (2010). Neuregulin-ErbB signalling promotes microglial proliferation and chemotaxis contributing to microgliosis and pain after peripheral nerve injury. *J. Neurosci.* 30 5437–5450 10.1523/JNEUROSCI.5169-09.2010 20392965 PMC2862659

[B34] Campos-Melo D. Droppelmann C. A. He Z. Volkening K. Strong M. J. (2013). Altered microRNA expression profile in Amyotrophic Lateral Sclerosis: a role in the regulation of NFL mRNA levels. *Mol. Brain* 6 26 10.1186/1756-6606-6-26 PMC3668997 23705811

[B35] Cardona A. E. Pioro E. P. Sasse M. E. Kostenko V. Cardona S. M. Dijkstra I. M. (2006). Control of microglial neurotoxicity by the fractalkine receptor. *Nat. Neurosci.* 9 917–924 10.1038/nn1715 16732273

[B36] Cardoso A. L. Guedes J. R. Pereira de Almeida L Pedroso De Lima M. C. (2012). miR-155 modulates microglia-mediated immune response by down-regulating SOCS-1 and promoting cytokine and nitric oxide production. *Immunology* 135 73–88 10.1111/j.1365-2567.2011.03514.x 22043967 PMC3246654

[B37] Cardoso F. L. Brites D. Brito M. A. (2010). Looking at the blood–brain barrier: molecular anatomy and possible investigation approaches. *Brain Res. Rev.* 64 328–363 10.1016/j.brainresrev.2010.05.003 20685221

[B38] Casoni F. Basso M. Massignan T. Gianazza E. Cheroni C. Salmona M. (2005). Protein nitration in a mouse model of familial amyotrophic lateral sclerosis: possible multifunctional role in the pathogenesis. *J. Biol. Chem.* 280 16295–16304 10.1074/jbc.M413111200 15699043

[B39] Casula M. Iyer A. M. Spliet W. G. Anink J. J. Steentjes K. Sta M. (2011). Toll-like receptor signalling in amyotrophic lateral sclerosis spinal cord tissue. *Neuroscience* 179 233–243 10.1016/j.neuroscience.2011.02.001 21303685

[B40] Cereda C. Cova E. Di Poto C. Galli A. Mazzini G. Corato M. (2006). Effect of nitric oxide on lymphocytes from sporadic amyotrophic lateral sclerosis patients: toxic or protective role? *Neurol. Sci.* 27 312–316 10.1007/s10072-006-0702-z 17122939

[B41] Chang C. Lang H. Geng N. Wang J. Li N. Wang X. (2013). Exosomes of BV-2 cells induced by alpha-synuclein: important mediator of neurodegeneration in PD. *Neurosci. Lett.* 548 190–195 10.1016/j.neulet.2013.06.009 23792198

[B42] Chapman G. A. Moores K. Harrison D. Campbell C. A. Stewart B. R. Strijbos P. J. (2000). Fractalkine cleavage from neuronal membranes represents an acute event in the inflammatory response to excitotoxic brain damage. *J. Neurosci.* 20 RC87 10.1523/JNEUROSCI.20-15-j0004.2000 PMC6772533 10899174

[B43] Chen L. C. Smith A. Ben Y. Zukic B. Ignacio S. Moore D. (2004). Temporal gene expression patterns in G93A/SOD1 mouse. *Amyotroph. Lateral Scler. Other Motor Neuron Disord.* 5 164–171 10.1080/14660820410017091 15512905

[B44] Chen S. Zhang X. Song L. Le W. (2012). Autophagy dysregulation in amyotrophic lateral sclerosis. *Brain Pathol.* 22 110–116 10.1111/j.1750-3639.2011.00546.x 22150926 PMC8029048

[B45] Chen S. C. Leach M. W. Chen Y. Cai X. Y. Sullivan L. Wiekowski M. (2002). Central nervous system inflammation and neurological disease in transgenic mice expressing the CC chemokine CCL21 in oligodendrocytes. *J. Immunol.* 168 1009–1017 11801633 10.4049/jimmunol.168.3.1009

[B46] Chhor V. Le Charpentier T. Lebon S. Ore M. V. Celador I. L. Josserand J. (2013). Characterization of phenotype markers and neuronotoxic potential of polarised primary microglia in vitro. *Brain Behav. Immun.* 32 70–85 10.1016/j.bbi.2013.02.005 23454862 PMC3694309

[B47] Chia R. Tattum M. H. Jones S. Collinge J. Fisher E. M. Jackson G. S. (2010). Superoxide dismutase 1 and tgSOD1 mouse spinal cord seed fibrils, suggesting a propagative cell death mechanism in amyotrophic lateral sclerosis. *PLoS ONE* 5:e10627 10.1371/journal.pone.0010627 PMC2869360 20498711

[B48] Chiu I. M. Chen A. Zheng Y. Kosaras B. Tsiftsoglou S. A. Vartanian T. K. (2008). T lymphocytes potentiate endogenous neuroprotective inflammation in a mouse model of ALS. *Proc. Natl. Acad. Sci. U.S.A.* 105 17913–17918 10.1073/pnas.0804610105 18997009 PMC2581614

[B49] Chiu I. M. Morimoto E. T. Goodarzi H. Liao J. T. O’Keeffe S. Phatnani H. P. (2013). A neurodegeneration-specific gene-expression signature of acutely isolated microglia from an amyotrophic lateral sclerosis mouse model. *Cell Rep.* 4 385–401 10.1016/j.celrep.2013.06.018 23850290 PMC4272581

[B50] Clark A. K. Malcangio M. (2012). Microglial signalling mechanisms: cathepsin S and fractalkine. *Exp. Neurol.* 234 283–292 10.1016/j.expneurol.2011.09.012 21946268

[B51] Clark A. K. Yip P. K. Grist J. Gentry C. Staniland A. A. Marchand F. (2007). Inhibition of spinal microglial cathepsin S for the reversal of neuropathic pain. *Proc. Natl. Acad. Sci. U.S.A.* 104 10655–10660 10.1073/pnas.0610811104 17551020 PMC1965568

[B52] Clark A. K. Yip P. K. Malcangio M. (2009). The liberation of fractalkine in the dorsal horn requires microglial cathepsin S. *J. Neurosci.* 29 6945–6954 10.1523/JNEUROSCI.0828-09.2009 19474321 PMC2698289

[B53] Clement A. M. Nguyen M. D. Roberts E. A. Garcia M. L. Boillee S. Rule M. (2003). Wild-type nonneuronal cells extend survival of SOD1 mutant motor neurons in ALS mice. *Science* 302 113–117 10.1126/science.1086071 14526083

[B54] Cook A. Hippensteel R. Shimizu S. Nicolai J. Fatatis A. Meucci O. (2010). Interactions between chemokines: regulation of fractalkine/CX3CL1 homeostasis by SDF/CXCL12 in cortical neurons. *J. Biol. Chem.* 285 10563–10571 10.1074/jbc.M109.035477 20124406 PMC2856264

[B55] Corcia P. Tauber C. Vercoullie J. Arlicot N. Prunier C. Praline J. (2012). Molecular imaging of microglial activation in amyotrophic lateral sclerosis. *PLoS ONE* 7:e52941 10.1371/journal.pone.0052941 PMC3534121 23300829

[B56] Cunha C. (2012). *Exploring Motor Neuron Degeneration in ALS – Prevention by Glycoursodeoxycholic Acid and Signalling to Microglia*. Master’s thesis, Faculdade de Ciências e Tecnologia, Universidade Nova de Lisboa, Lisbon

[B57] Czirr E. Wyss-Coray T. (2012). The immunology of neurodegeneration. *J. Clin. Invest.* 122 1156–1163 10.1172/JCI58656 22466657 PMC3315444

[B58] David S. Kroner A. (2011). Repertoire of microglial and macrophage responses after spinal cord injury. *Nat. Rev. Neurosci.* 12 388–399 10.1038/nrn3053 21673720

[B59] Davoust N. Vuaillat C. Androdias G. Nataf S. (2008). From bone marrow to microglia: barriers and avenues. *Trends Immunol.* 29 227–234 10.1016/j.it.2008.01.010 18396103

[B60] Deepa P. Shahani N. Alladi P. A. Vijayalakshmi K. Sathyaprabha T. N. Nalini A. (2011). Down regulation of trophic factors in neonatal rat spinal cord after administration of cerebrospinal fluid from sporadic amyotrophic lateral sclerosis patients. *J. Neural. Transm.* 118 531–538 10.1007/s00702-010-0520-6 21069391

[B61] Deiva K. Geeraerts T. Salim H. Leclerc P. Hery C. Hugel B. (2004). Fractalkine reduces *N*-methyl-D-aspartate-induced calcium flux and apoptosis in human neurons through extracellular signal-regulated kinase activation. *Eur. J. Neurosci.* 20 3222–3232 10.1111/j.1460-9568.2004.03800.x 15610155

[B62] DeJesus-Hernandez M. Mackenzie I. R. Boeve B. F. Boxer A. L. Baker M. Rutherford N. J. (2011). Expanded GGGGCC hexanucleotide repeat in noncoding region of C9ORF72 causes chromosome 9p-linked FTD and ALS. *Neuron* 72 245–256 10.1016/j.neuron.2011.09.011 21944778 PMC3202986

[B63] de Jong E. K. Dijkstra I. M. Hensens M. Brouwer N. Van Amerongen M. Liem R. S. (2005). Vesicle-mediated transport and release of CCL21 in endangered neurons: a possible explanation for microglia activation remote from a primary lesion. *J. Neurosci.* 25 7548–7557 10.1523/JNEUROSCI.1019-05.2005 16107642 PMC6725403

[B64] Demestre M. Parkin-Smith G. Petzold A. Pullen A. H. (2005). The pro and the active form of matrix metalloproteinase-9 is increased in serum of patients with amyotrophic lateral sclerosis. *J. Neuroimmunol.* 159 146–154 10.1016/j.jneuroim.2004.09.015 15652414

[B65] Desforges N. M. Hebron M. L. Algarzae N. K. Lonskaya I. Moussa C. E. (2012). Fractalkine mediates communication between pathogenic proteins and microglia: implications of anti-inflammatory treatments in different stages of neurodegenerative diseases. *Int. J. Alzheimers Dis.* 2012 345472 10.1155/2012/345472 PMC3420133 22919540

[B66] Diaz-Amarilla P. Olivera-Bravo S. Trias E. Cragnolini A. Martinez-Palma L. Cassina P. (2011). Phenotypically aberrant astrocytes that promote motoneuron damage in a model of inherited amyotrophic lateral sclerosis. *Proc. Natl. Acad. Sci. U.S.A.* 108 18126–18131 10.1073/pnas.1110689108 22010221 PMC3207668

[B67] Dibaj P. Steffens H. Zschuntzsch J. Kirchhoff F. Schomburg E. D. Neusch C. (2011). In vivo imaging reveals rapid morphological reactions of astrocytes towards focal lesions in an ALS mouse model. *Neurosci. Lett.* 497 148–151 10.1016/j.neulet.2011.04.049 21539893

[B68] Dittel B. N. (2008). CD4 T cells: balancing the coming and going of autoimmune-mediated inflammation in the CNS. *Brain Behav. Immun.* 22 421–430 10.1016/j.bbi.2007.11.010 18207698 PMC2376206

[B69] Di Virgilio F. (2000). Dr. Jekyll/Mr. Hyde: the dual role of extracellular ATP. *J. Auton. Nerv. Syst.* 81 59–63 10.1016/S0165-1838(00)00114-4 10869701

[B70] Drechsel D. A. Estevez A. G. Barbeito L. Beckman J. S. (2012). Nitric oxide-mediated oxidative damage and the progressive demise of motor neurons in ALS. *Neurotox. Res.* 22 251–264 10.1007/s12640-012-9322-y 22488161 PMC4145402

[B71] El Andaloussi S. Lakhal S. Mager I. Wood M. J. (2013). Exosomes for targeted siRNA delivery across biological barriers. *Adv. Drug Deliv. Rev.* 65 391–397 10.1016/j.addr.2012.08.008 22921840

[B72] Elliott J. L. (1999). Experimental models of amyotrophic lateral sclerosis. *Neurobiol. Dis.* 6 310–320 10.1006/nbdi.1999.0266 10527800

[B73] Enokido Y. Yoshitake A. Ito H. Okazawa H. (2008). Age-dependent change of HMGB1 and DNA double-strand break accumulation in mouse brain. *Biochem. Biophys. Res. Commun.* 376 128–133 10.1016/j.bbrc.2008.08.108 18762169

[B74] Evans M. C. Couch Y. Sibson N. Turner M. R. (2013). Inflammation and neurovascular changes in amyotrophic lateral sclerosis. *Mol. Cell. Neurosci.* 53 34–41 10.1016/j.mcn.2012.10.008 23110760

[B75] Fang P. Schachner M. Shen Y. Q. (2012). HMGB1 in development and diseases of the central nervous system. *Mol. Neurobiol.* 45 499–506 10.1007/s12035-012-8264-y 22580958

[B76] Fendrick S. E. Xue Q. S. Streit W. J. (2007). Formation of multinucleated giant cells and microglial degeneration in rats expressing a mutant Cu/Zn superoxide dismutase gene. *J. Neuroinflammation* 4 9 10.1186/1742-2094-4-9 PMC1808448 17328801

[B77] Ferraiuolo L. Higginbottom A. Heath P. R. Barber S. Greenald D. Kirby J. (2011). Dysregulation of astrocyte-motoneuron cross-talk in mutant superoxide dismutase 1-related amyotrophic lateral sclerosis. *Brain* 134 2627–2641 10.1093/brain/awr193 21908873 PMC3170534

[B78] Fiszman M. L. Borodinsky L. N. Ricart K. C. Sanz O. P. Sica R. E. (1999). Cu/Zn superoxide dismutase activity at different ages in sporadic amyotrophic lateral sclerosis. *J. Neurol. Sci.* 162 34–37 10.1016/S0022-510X(98)00272-X 10064166

[B79] Fitzner D. Schnaars M. Van Rossum D. Krishnamoorthy G. Dibaj P. Bakhti M. (2011). Selective transfer of exosomes from oligodendrocytes to microglia by macropinocytosis. *J. Cell Sci.* 124 447–458 10.1242/jcs.074088 21242314

[B80] Frakes A. E. Ferraiuolo L. Haidet-Phillips A. M. Schmelzer L. Braun L. Miranda C. J. (2014). Microglia induce motor neuron death via the classical NF-kappaB pathway in amyotrophic lateral sclerosis. *Neuron* 81 1009–1023 10.1016/j.neuron.2014.01.013 24607225 PMC3978641

[B81] Fricker M. Oliva-Martin M. J. Brown G. C. (2012a). Primary phagocytosis of viable neurons by microglia activated with LPS or Abeta is dependent on calreticulin/LRP phagocytic signalling. *J. Neuroinflammation* 9 196 10.1186/1742-2094-9-196 PMC3481398 22889139

[B82] Fricker M. Neher J. J. Zhao J. W. Thery C. Tolkovsky A. M. Brown G. C. (2012b). MFG-E8 mediates primary phagocytosis of viable neurons during neuroinflammation. *J. Neurosci.* 32 2657–2666 10.1523/JNEUROSCI.4837-11.2012 22357850 PMC3312099

[B83] Fruhbeis C. Frohlich D. Kramer-Albers E. M. (2012). Emerging roles of exosomes in neuron–glia communication. *Front. Physiol.* 3:119 10.3389/fphys.2012.00119 PMC3339323 22557979

[B84] Furukawa Y. Kaneko K. Watanabe S. Yamanaka K. Nukina N. (2013). Intracellular seeded aggregation of mutant Cu,Zn-superoxide dismutase associated with amyotrophic lateral sclerosis. *FEBS Lett.* 587 2500–2505 10.1016/j.febslet.2013.06.046 23831581

[B85] Gandelman M. Peluffo H. Beckman J. S. Cassina P. Barbeito L. (2010). Extracellular ATP and the P2X7 receptor in astrocyte-mediated motor neuron death: implications for amyotrophic lateral sclerosis. *J. Neuroinflammation* 7 33 10.1186/1742-2094-7-33 PMC2901222 20534165

[B86] Gao H. M. Zhou H. Zhang F. Wilson B. C. Kam W. Hong J. S. (2011). HMGB1 acts on microglia Mac1 to mediate chronic neuroinflammation that drives progressive neurodegeneration. *J. Neurosci.* 31 1081–1092 10.1523/JNEUROSCI.3732-10.2011 21248133 PMC3046932

[B87] Garbuzova-Davis S. Haller E. Saporta S. Kolomey I. Nicosia S. V. Sanberg P. R. (2007). Ultrastructure of blood–brain barrier and blood-spinal cord barrier in SOD1 mice modeling ALS. *Brain Res.* 1157 126–137 10.1016/j.brainres.2007.04.044 17512910

[B88] Garbuzova-Davis S. Rodrigues M. C. Mirtyl S. Turner S. Mitha S. Sodhi J. (2012). Multiple intravenous administrations of human umbilical cord blood cells benefit in a mouse model of ALS. *PLoS ONE* 7:e31254 10.1371/journal.pone.0031254 PMC3272008 22319620

[B89] Garton K. J. Gough P. J. Blobel C. P. Murphy G. Greaves D. R. Dempsey P. J. (2001). Tumor necrosis factor-alpha-converting enzyme (ADAM17) mediates the cleavage and shedding of fractalkine (CX3CL1). *J. Biol. Chem.* 276 37993–38001 10.1074/jbc.M106434200 11495925

[B90] Gerber Y. N. Sabourin J. C. Rabano M. Vivanco M. Perrin F. E. (2012). Early functional deficit and microglial disturbances in a mouse model of amyotrophic lateral sclerosis. *PLoS ONE* 7:e36000 10.1371/journal.pone.0036000 PMC3338492 22558300

[B91] Glass C. K. Saijo K. Winner B. Marchetto M. C. Gage F. H. (2010). Mechanisms underlying inflammation in neurodegeneration. *Cell* 140 918–934 10.1016/j.cell.2010.02.016 20303880 PMC2873093

[B92] Gomes C. Keller S. Altevogt P. Costa J. (2007). Evidence for secretion of Cu,Zn superoxide dismutase via exosomes from a cell model of amyotrophic lateral sclerosis. *Neurosci. Lett.* 428 43–46 10.1016/j.neulet.2007.09.024 17942226

[B93] Gomez-Pinedo U. Yanez M. Matias-Guiu J. Galan L. Guerrero-Sola A. Benito-Martin M. S. (2013). Cellular changes in motor neuron cell culture produced by cytotoxic cerebrospinal fluid from patients with amyotrophic lateral sclerosis. *Neurologia.* 10.1016/j.nrl.2013.08.001 [Epub ahead of print] 24144827

[B94] Gonzalez de Aguilar J. L. Niederhauser-Wiederkehr C. Halter B. De Tapia M. Di Scala F. Demougin P. (2008). Gene profiling of skeletal muscle in an amyotrophic lateral sclerosis mouse model. *Physiol. Genomics* 32 207–218 10.1152/physiolgenomics.00017.2007 18000159

[B95] Gottschall P. E. Deb S. (1996). Regulation of matrix metalloproteinase expressions in astrocytes, microglia and neurons. *Neuroimmunomodulation* 3 69–75 10.1159/000097229 8945720

[B96] Gough P. J. Garton K. J. Wille P. T. Rychlewski M. Dempsey P. J. Raines E. W. (2004). A disintegrin and metalloproteinase 10-mediated cleavage and shedding regulates the cell surface expression of CXC chemokine ligand 16. *J. Immunol.* 172 3678–3685 10.4049/jimmunol.172.6.3678 15004171

[B97] Grad L. I. Yerbury J. J. Turner B. J. Guest W. C. Pokrishevsky E. O’neill M. A. (2014). Intercellular propagated misfolding of wild-type Cu/Zn superoxide dismutase occurs via exosome-dependent and -independent mechanisms. Proc. *Natl. Acad. Sci. U.S.A.* 111 3620–3625 10.1073/pnas.1312245111 PMC3948312 24550511

[B98] Grosskreutz J. Van Den Bosch L. Keller B. U. (2010). Calcium dysregulation in amyotrophic lateral sclerosis. *Cell Calcium* 47 165–174 10.1016/j.ceca.2009.12.002 20116097

[B99] Guegan C. Vila M. Rosoklija G. Hays A. P. Przedborski S. (2001). Recruitment of the mitochondrial-dependent apoptotic pathway in amyotrophic lateral sclerosis. *J. Neurosci.* 21 6569–6576 11517246 10.1523/JNEUROSCI.21-17-06569.2001 PMC6763092

[B100] Gurney M. E. Pu H. Chiu A. Y. Dal Canto M. C. Polchow C. Y. Alexander D. D. (1994). Motor neuron degeneration in mice that express a human Cu,Zn superoxide dismutase mutation. *Science* 264 1772–1775 10.1126/science.8209258 8209258

[B101] Hadass O. Tomlinson B. N. Gooyit M. Chen S. Purdy J. J. Walker J. M. (2013). Selective inhibition of matrix metalloproteinase-9 attenuates secondary damage resulting from severe traumatic brain injury. *PLoS ONE* 8:e76904 10.1371/journal.pone.0076904 PMC3806745 24194849

[B102] Haidet-Phillips A. M. Hester M. E. Miranda C. J. Meyer K. Braun L. Frakes A. (2011). Astrocytes from familial and sporadic ALS patients are toxic to motor neurons. *Nat. Biotechnol.* 29 824–828 10.1038/nbt.1957 21832997 PMC3170425

[B103] Haramati S. Chapnik E. Sztainberg Y. Eilam R. Zwang R. Gershoni N. (2010). miRNA malfunction causes spinal motor neuron disease. *Proc. Natl. Acad. Sci. U.S.A.* 107 13111–13116 10.1073/pnas.1006151107 20616011 PMC2919953

[B104] Harrison J. K. Jiang Y. Chen S. Xia Y. Maciejewski D. Mcnamara R. K. (1998). Role for neuronally derived fractalkine in mediating interactions between neurons and CX3BC1-expressing microglia. *Proc. Natl. Acad. Sci. U.S.A.* 95 10896–10901 10.1073/pnas.95.18.10896 9724801 PMC27992

[B105] Hatori K. Nagai A. Heisel R. Ryu J. K. Kim S. U. (2002). Fractalkine and fractalkine receptors in human neurons and glial cells. *J. Neurosci. Res.* 69 418–426 10.1002/jnr.10304 12125082

[B106] He Q. You H. Li X. M. Liu T. H. Wang P. Wang B. E. (2012). HMGB1 promotes the synthesis of pro-IL-1beta and pro-IL-18 by activation of p38 MAPK and NF-kappaB through receptors for advanced glycation end-products in macrophages. *Asian Pac. J. Cancer Prev.* 13 1365–1370 10.7314/APJCP.2012.13.4.1365 22799333

[B107] He X. Zhang L. Yao X. Hu J. Yu L. Jia H. (2013). Association studies of MMP-9 in Parkinson’s disease and amyotrophic lateral sclerosis. *PLoS ONE* 8:e73777 10.1371/journal.pone.0073777 PMC3767588 24040066

[B108] Heath P. R. Kirby J. Shaw P. J. (2013). Investigating cell death mechanisms in amyotrophic lateral sclerosis using transcriptomics. *Front. Cell. Neurosci.* 7:259 10.3389/fncel.2013.00259 PMC3865770 24381542

[B109] Henkel J. S. Beers D. R. Zhao W. Appel S. H. (2009). Microglia in ALS: the good, the bad, and the resting. *J. Neuroimmune Pharmacol.* 4 389–398 10.1007/s11481-009-9171-5 19731042

[B110] Hinze A. Stolzing A. (2011). Differentiation of mouse bone marrow derived stem cells toward microglia-like cells. *BMC Cell Biol.* 12:35 10.1186/1471-2121-12-35 PMC3175184 21854582

[B111] Hoelzinger D. B. Nakada M. Demuth T. Rosensteel T. Reavie L. B. Berens M. E. (2008). Autotaxin: a secreted autocrine/paracrine factor that promotes glioma invasion. *J. Neurooncol.* 86 297–309 10.1007/s11060-007-9480-6 17928955

[B112] Holm T. H. Draeby D. Owens T. (2012). Microglia are required for astroglial Toll-like receptor 4 response and for optimal TLR2 and TLR3 response. *Glia* 60 630–638 10.1002/glia.22296 22271465

[B113] Holmoy T. (2008). T cells in amyotrophic lateral sclerosis. *Eur. J. Neurol.* 15 360–366 10.1111/j.1468-1331.2008.02065.x 18266871

[B114] Hooper C. Sainz-Fuertes R. Lynham S. Hye A. Killick R. Warley A. (2012). Wnt3a induces exosome secretion from primary cultured rat microglia. *BMC Neurosci.* 13:144 10.1186/1471-2202-13-144 PMC3541220 23173708

[B115] Ilieva H. Polymenidou M. Cleveland D. W. (2009). Non-cell autonomous toxicity in neurodegenerative disorders: ALS and beyond. *J. Cell Biol.* 187 761–772 10.1083/jcb.200908164 19951898 PMC2806318

[B116] Ince P. G. Highley J. R. Kirby J. Wharton S. B. Takahashi H. Strong M. J. (2011). Molecular pathology and genetic advances in amyotrophic lateral sclerosis: an emerging molecular pathway and the significance of glial pathology. *Acta Neuropathol.* 122 657–671 10.1007/s00401-011-0913-0 22105541

[B117] Inoue H. Tsukita K. Iwasato T. Suzuki Y. Tomioka M. Tateno M. (2003). The crucial role of caspase-9 in the disease progression of a transgenic ALS mouse model. *EMBO J.* 22 6665–6674 10.1093/emboj/cdg634 14657037 PMC291829

[B118] Jiang M. Xiang Y. Wang D. Gao J. Liu D. Liu Y. (2012). Dysregulated expression of miR-146a contributes to age-related dysfunction of macrophages. *Aging Cell* 11 29–40 10.1111/j.1474-9726.2011.00757.x 21981419

[B119] Julien J. P. (2001). Amyotrophic lateral sclerosis. unfolding the toxicity of the misfolded. *Cell* 104 581–591 10.1016/S0092-8674(01)00244-6 11239414

[B120] Jung S. Schwartz M. (2012). Non-identical twins – microglia and monocyte-derived macrophages in acute injury and autoimmune inflammation. *Front. Immunol.* 3:89 10.3389/fimmu.2012.00089 PMC3345364 22566968

[B121] Jurgens H. A. Johnson R. W. (2012). Dysregulated neuronal-microglial cross-talk during aging, stress and inflammation. *Exp. Neurol.* 233 40–48 10.1016/j.expneurol.2010.11.014 21110971 PMC3071456

[B122] Kang S. H. Fukaya M. Yang J. K. Rothstein J. D. Bergles D. E. (2010). NG2^+^ CNS glial progenitors remain committed to the oligodendrocyte lineage in postnatal life and following neurodegeneration. *Neuron* 68 668–681 10.1016/j.neuron.2010.09.009 21092857 PMC2989827

[B123] Kang S. H. Li Y. Fukaya M. Lorenzini I. Cleveland D. W. Ostrow L. W. (2013). Degeneration and impaired regeneration of gray matter oligodendrocytes in amyotrophic lateral sclerosis. *Nat. Neurosci.* 16 571–579 10.1038/nn.3357 23542689 PMC3637847

[B124] Kaplan A. Spiller K. J. Towne C. Kanning K. C. Choe G. T. Geber A. (2014). Neuronal matrix metalloproteinase-9 is a determinant of selective neurodegeneration. *Neuron* 81 333–348 10.1016/j.neuron.2013.12.009 24462097 PMC6015650

[B125] Kaspar B. K. Llado J. Sherkat N. Rothstein J. D. Gage F. H. (2003). Retrograde viral delivery of IGF-1 prolongs survival in a mouse ALS model. *Science* 301 839–842 10.1126/science.1086137 12907804

[B126] Kato S. (2008). Amyotrophic lateral sclerosis models and human neuropathology: similarities and differences. *Acta Neuropathol.* 115 97–114 10.1007/s00401-007-0308-4 18026741

[B127] Kawabata H. Setoguchi T. Yone K. Souda M. Yoshida H. Kawahara K. (2010). High mobility group box 1 is upregulated after spinal cord injury and is associated with neuronal cell apoptosis. *Spine (Phila Pa 1976)* 35 1109–1115 10.1097/BRS.0b013e3181bd14b6 20195207

[B128] Keller A. F. Gravel M. Kriz J. (2009). Live imaging of amyotrophic lateral sclerosis pathogenesis: disease onset is characterized by marked induction of GFAP in Schwann cells. *Glia* 57 1130–1142 10.1002/glia.20836 19115383

[B129] Kiernan M. C. (2014). ALS and neuromuscular disease: in search of the Holy Grail. *Lancet Neurol.* 13 13–14 10.1016/S1474-4422(13)70226-6 24331786

[B130] Kiernan M. C. Vucic S. Cheah B. C. Turner M. R. Eisen A. Hardiman O. (2011). Amyotrophic lateral sclerosis. *Lancet* 377 942–955 10.1016/S0140-6736(10)61156-7 21296405

[B131] Kim H. Cooke M. J. Shoichet M. S. (2012). Creating permissive microenvironments for stem cell transplantation into the central nervous system. *Trends Biotechnol.* 30 55–63 10.1016/j.tibtech.2011.07.002 21831464

[B132] Kim J. B. Sig Choi J. Yu Y. M. Nam K. Piao C. S. Kim S. W. (2006a). HMGB1, a novel cytokine-like mediator linking acute neuronal death and delayed neuroinflammation in the postischemic brain. *J. Neurosci.* 26 6413–6421 10.1523/JNEUROSCI.3815-05.2006 16775128 PMC6674036

[B133] Kim Y. S. Martinez T. Deshpande D. M. Drummond J. Provost-Javier K. Williams A. (2006b). Correction of humoral derangements from mutant superoxide dismutase 1 spinal cord. *Ann. Neurol.* 60 716–728 10.1002/ana.21034 17192933

[B134] Kim J. M. Noh E. M. Kwon K. B. Kim J. S. You Y. O. Hwang J. K. (2013). Suppression of TPA-induced tumor cell invasion by sulfuretin via inhibition of NF-kappaB-dependent MMP-9 expression. *Oncol. Rep.* 29 1231–1237 10.3892/or.2012.2218 23292685

[B135] Konnecke H. Bechmann I. (2013). The role of microglia and matrix metalloproteinases involvement in neuroinflammation and gliomas. *Clin. Dev. Immunol.* 2013 914104 10.1155/2013/914104 PMC3759277 24023566

[B136] Koval E. D. Shaner C. Zhang P. Du Maine X. Fischer K. Tay J. (2013). Method for widespread microRNA-155 inhibition prolongs survival in ALS-model mice. *Hum. Mol. Genet.* 22 4127–4135 10.1093/hmg/ddt261 23740943 PMC3781640

[B137] Kushner P. D. Stephenson D. T. Wright S. (1991). Reactive astrogliosis is widespread in the subcortical white matter of amyotrophic lateral sclerosis brain. *J. Neuropathol. Exp. Neurol.* 50 263–277 10.1097/00005072-199105000-00008 2022968

[B138] Lagier-Tourenne C. Cleveland D. W. (2009). Rethinking ALS: the FUS about TDP-43. *Cell* 136 1001–1004 10.1016/j.cell.2009.03.006 19303844 PMC3110083

[B139] Lange S. Trost A. Tempfer H. Bauer H. C. Bauer H. Rohde E. (2012). Brain pericyte plasticity as a potential drug target in CNS repair. *Drug Discov. Today* 18 456–463 10.1016/j.drudis.2012.12.007 23266366

[B140] Lasiene J. Yamanaka K. (2011). Glial cells in amyotrophic lateral sclerosis. *Neurol. Res. Int.* 2011 718987 10.1155/2011/718987 PMC3135155 21766027

[B141] Lee C. T. Chiu Y. W. Wang K. C. Hwang C. S. Lin K. H. Lee I. T. (2013a). Riluzole and prognostic factors in amyotrophic lateral sclerosis long-term and short-term survival: a population-based study of 1149 cases in Taiwan. *J. Epidemiol.* 23 35–40 10.2188/jea.JE20120119 23117224 PMC3700231

[B142] Lee H. Lee S. Cho I. H. Lee S. J. (2013b). Toll-like receptors: sensor molecules for detecting damage to the nervous system. *Curr. Protein Pept. Sci.* 14 33–42 10.2174/1389203711314010006 23441900

[B143] Lee J. C. Seong J. Kim S. H. Lee S. J. Cho Y. J. An J. (2012). Replacement of microglial cells using clodronate liposome and bone marrow transplantation in the central nervous system of SOD1(G93A) transgenic mice as an in vivo model of amyotrophic lateral sclerosis. *Biochem. Biophys. Res. Commun.* 418 359–365 10.1016/j.bbrc.2012.01.026 22269142

[B144] Leinster V. H. Joy M. T. Vuononvirta R. E. Bolsover S. R. Anderson P. N. (2013). ErbB1 epidermal growth factor receptor is a valid target for reducing the effects of multiple inhibitors of axonal regeneration. *Exp. Neurol.* 239 82–90 10.1016/j.expneurol.2012.09.007 23022459 PMC3556781

[B145] Leonardi-Essmann F. Emig M. Kitamura Y. Spanagel R. Gebicke-Haerter P. J. (2005). Fractalkine-upregulated milk-fat globule EGF factor-8 protein in cultured rat microglia. *J. Neuroimmunol.* 160 92–101 10.1016/j.jneuroim.2004.11.012 15710462

[B146] Le Pichon C. E. Dominguez S. L. Solanoy H. Ngu H. Lewin-Koh N. Chen M. (2013). EGFR inhibitor erlotinib delays disease progression but does not extend survival in the SOD1 mouse model of ALS. *PLoS ONE* 8:e62342 10.1371/journal.pone.0062342 PMC3637182 23638043

[B147] Lepore A. C. O’Donnell J. Kim A. S. Williams T. Tuteja A. Rao M. S. (2011). Human glial-restricted progenitor transplantation into cervical spinal cord of the SOD1 mouse model of ALS. *PLoS ONE* 6:e25968 10.1371/journal.pone.0025968 PMC3187829 21998733

[B148] Lepore A. C. Rauck B. Dejea C. Pardo A. C. Rao M. S. Rothstein J. D. (2008). Focal transplantation-based astrocyte replacement is neuroprotective in a model of motor neuron disease. *Nat. Neurosci.* 11 1294–1301 10.1038/nn.2210 18931666 PMC2656686

[B149] Le Verche V. Ikiz B. Jacquier A. Przedborski S. Re D. B. (2011). Glutamate pathway implication in amyotrophic lateral sclerosis: what is the signal in the noise? *J. Receptor Ligand Channel Res.* 4 1–22 10.2147/JRLCR.S6504

[B150] Lewis C. A. Manning J. Rossi F. Krieger C. (2012). The neuroinflammatory response in ALS: the roles of microglia and T cells. *Neurol. Res. Int.* 2012 803701 10.1155/2012/803701 PMC3362167 22666587

[B151] Lewis C. A. Solomon J. N. Rossi F. M. Krieger C. (2009). Bone marrow-derived cells in the central nervous system of a mouse model of amyotrophic lateral sclerosis are associated with blood vessels and express CX(3)BC1. *Glia* 57 1410–1419 10.1002/glia.20859 19243075

[B152] Li Y. R. King O. D. Shorter J. Gitler A. D. (2013). Stress granules as crucibles of ALS pathogenesis. *J. Cell Biol.* 201 361–372 10.1083/jcb.201302044 23629963 PMC3639398

[B153] Liao B. Zhao W. Beers D. R. Henkel J. S. Appel S. H. (2012). Transformation from a neuroprotective to a neurotoxic microglial phenotype in a mouse model of ALS. *Exp. Neurol.* 237 147–152 10.1016/j.expneurol.2012.06.011 22735487 PMC4126417

[B154] Liblau R. S. Gonzalez-Dunia D. Wiendl H. Zipp F. (2013). Neurons as targets for T cells in the nervous system. *Trends Neurosci.* 36 315–324 10.1016/j.tins.2013.01.008 23478065

[B155] Lilo E. Wald-Altman S. Solmesky L. J. Ben Yaakov K. Gershoni-Emek N. Bulvik S. (2013). Characterization of human sporadic ALS biomarkers in the familial ALS transgenic mSOD1(G93A) mouse model. *Hum. Mol. Genet.* 22 4720–4725 10.1093/hmg/ddt325 23836781

[B156] Lino M. M. Schneider C. Caroni P. (2002). Accumulation of SOD1 mutants in postnatal motoneurons does not cause motoneuron pathology or motoneuron disease. *J. Neurosci.* 22 4825–4832 12077179 10.1523/JNEUROSCI.22-12-04825.2002 PMC6757755

[B157] Liu D. Wen J. Liu J. Li L. (1999). The roles of free radicals in amyotrophic lateral sclerosis: reactive oxygen species and elevated oxidation of protein, DNA, and membrane phospholipids. *FASEB J.* 13 2318–2328 10593879 10.1096/fasebj.13.15.2318

[B158] Liu R. Li B. Flanagan S. W. Oberley L. W. Gozal D. Qiu M. (2002). Increased mitochondrial antioxidative activity or decreased oxygen free radical propagation prevent mutant SOD1-mediated motor neuron cell death and increase amyotrophic lateral sclerosis-like transgenic mouse survival. *J. Neurochem.* 80 488–500 10.1046/j.0022-3042.2001.00720.x 11905995

[B159] Liu Y. Hao W. Dawson A. Liu S. Fassbender K. (2009). Expression of amyotrophic lateral sclerosis-linked SOD1 mutant increases the neurotoxic potential of microglia via TLR2. *J. Biol. Chem.* 284 3691–3699 10.1074/jbc.M804446200 19091752

[B160] Liu Y. C. Chiang P. M. Tsai K. J. (2013). Disease animal models of TDP-43 proteinopathy and their pre-clinical applications. *Int. J. Mol. Sci.* 14 20079–20111 10.3390/ijms141020079 24113586 PMC3821604

[B161] Lobsiger C. S. Boillee S. Mcalonis-Downes M. Khan A. M. Feltri M. L. Yamanaka K. (2009). Schwann cells expressing dismutase active mutant SOD1 unexpectedly slow disease progression in ALS mice. *Proc. Natl. Acad. Sci. U.S.A.* 106 4465–4470 10.1073/pnas.0813339106 19251638 PMC2657393

[B162] Lobsiger C. S. Cleveland D. W. (2007). Glial cells as intrinsic components of non-cell-autonomous neurodegenerative disease. *Nat. Neurosci.* 10 1355–1360 10.1038/nn1988 17965655 PMC3110080

[B163] Lo Coco D. Veglianese P. Allievi E. Bendotti C. (2007). Distribution and cellular localization of high mobility group box protein 1 (HMGB1) in the spinal cord of a transgenic mouse model of ALS. *Neurosci. Lett.* 412 73–77 10.1016/j.neulet.2006.10.063 17196331

[B164] London A. Cohen M. Schwartz M. (2013). Microglia and monocyte-derived macrophages: functionally distinct populations that act in concert in CNS plasticity and repair. *Front. Cell. Neurosci.* 7:34 10.3389/fncel.2013.00034 PMC3625831 23596391

[B165] Lorenzl S. Narr S. Angele B. Krell H. W. Gregorio J. Kiaei M. (2006). The matrix metalloproteinases inhibitor Ro 28-2653 [correction of Ro 26-2853] extends survival in transgenic ALS mice. *Exp. Neurol.* 200 166–171 10.1016/j.expneurol.2006.01.026 16516196

[B166] Lu B. Wang H. Andersson U. Tracey K. J. (2013). Regulation of HMGB1 release by inflammasomes. *Protein Cell* 4 163–167 10.1007/s13238-012-2118-2 23483477 PMC4533838

[B167] Luo X. G. Chen S. D. (2012). The changing phenotype of microglia from homeostasis to disease. *Transl. Neurodegener.* 1 9 10.1186/2047-9158-1-9 PMC3514090 23210447

[B168] Luo X. G. Ding J. Q. Chen S. D. (2010). Microglia in the aging brain: relevance to neurodegeneration. *Mol. Neurodegener.* 5 12 10.1186/1750-1326-5-12 PMC2852379 20334662

[B169] Lyons A. Lynch A. M. Downer E. J. Hanley R. O’Sullivan J. B. Smith A. (2009). Fractalkine-induced activation of the phosphatidylinositol-3 kinase pathway attenuates microglial activation in vivo and in vitro. *J. Neurochem.* 110 1547–1556 10.1111/j.1471-4159.2009.06253.x 19627440

[B170] Magrane J. Hervias I. Henning M. S. Damiano M. Kawamata H. Manfredi G. (2009). Mutant SOD1 in neuronal mitochondria causes toxicity and mitochondrial dynamics abnormalities. *Hum. Mol. Genet.* 18 4552–4564 10.1093/hmg/ddp421 19779023 PMC2773270

[B171] Magrane J. Manfredi G. (2009). Mitochondrial function, morphology, and axonal transport in amyotrophic lateral sclerosis. *Antioxid. Redox Signal.* 11 1615–1626 10.1089/ARS.2009.2604 19344253 PMC2789440

[B172] Matsumoto H. Kumon Y. Watanabe H. Ohnishi T. Shudou M. Ii C. (2007). Antibodies to CD11b, CD68, and lectin label neutrophils rather than microglia in traumatic and ischemic brain lesions. *J. Neurosci. Res.* 85 994–1009 10.1002/jnr.21198 17265469

[B173] Meme W. Calvo C. F. Froger N. Ezan P. Amigou E. Koulakoff A. (2006). Proinflammatory cytokines released from microglia inhibit gap junctions in astrocytes: potentiation by beta-amyloid. *FASEB J.* 20 494–496 10.1096/fj.05-4297fje 16423877

[B174] Mimoto T. Miyazaki K. Morimoto N. Kurata T. Satoh K. Ikeda Y. (2012). Impaired antioxidative Keap1/Nrf2 system and the downstream stress protein responses in the motor neuron of ALS model mice. *Brain Res.* 1446 109–118 10.1016/j.brainres.2011.12.064 22353756

[B175] Min J. H. Hong Y. H. Sung J. J. Kim S. M. Lee J. B. Lee K. W. (2012). Oral solubilized ursodeoxycholic acid therapy in amyotrophic lateral sclerosis: a randomized cross-over trial. *J. Korean Med. Sci.* 27 200–206 10.3346/jkms.2012.27.2.200 22323869 PMC3271295

[B176] Miyazaki K. Ohta Y. Nagai M. Morimoto N. Kurata T. Takehisa Y. (2011). Disruption of neurovascular unit prior to motor neuron degeneration in amyotrophic lateral sclerosis. *J. Neurosci. Res.* 89 718–728 10.1002/jnr.22594 21337372

[B177] Mizuno T. Kawanokuchi J. Numata K. Suzumura A. (2003). Production and neuroprotective functions of fractalkine in the central nervous system. *Brain Res.* 979 65–70 10.1016/S0006-8993(03)02867-1 12850572

[B178] Morel L. Regan M. Higashimori H. Ng S. K. Esau C. Vidensky S. (2013). Neuronal exosomal miRNA-dependent translational regulation of astroglial glutamate transporter GLT1. *J. Biol. Chem.* 288 7105–7116 10.1074/jbc.M112.410944 23364798 PMC3591620

[B179] Moreno-Lopez B. Sunico C. R. Gonzalez-Forero D. (2011). NO orchestrates the loss of synaptic boutons from adult “sick” motoneurons: modeling a molecular mechanism. *Mol. Neurobiol.* 43 41–66 10.1007/s12035-010-8159-8 21190141

[B180] Mori A. Yamashita S. Uchino K. Suga T. Ikeda T. Takamatsu K. (2011). Derlin-1 overexpression ameliorates mutant SOD1-induced endoplasmic reticulum stress by reducing mutant SOD1 accumulation. *Neurochem. Int.* 58 344–353 10.1016/j.neuint.2010.12.010 21185345

[B181] Munch C. O’Brien J. Bertolotti A. (2011). Prion-like propagation of mutant superoxide dismutase-1 misfolding in neuronal cells. *Proc. Natl. Acad. Sci. U.S.A.* 108 3548–3553 10.1073/pnas.1017275108 21321227 PMC3048161

[B182] Nakanishi H. (2003). Neuronal and microglial cathepsins in aging and age-related diseases. *Ageing Res. Rev.* 2 367–381 10.1016/S1568-1637(03)00027-8 14522241

[B183] Nakanishi H. Wu Z. (2009). Microglia-aging: roles of microglial lysosome- and mitochondria-derived reactive oxygen species in brain aging. *Behav. Brain Res.* 201 1–7 10.1016/j.bbr.2009.02.001 19428609

[B184] Nardo G. Pozzi S. Pignataro M. Lauranzano E. Spano G. Garbelli S. (2011). Amyotrophic lateral sclerosis multiprotein biomarkers in peripheral blood mononuclear cells. *PLoS ONE* 6:e25545 10.1371/journal.pone.0025545 PMC3187793 21998667

[B185] Neusch C. Bahr M. Schneider-Gold C. (2007). Glia cells in amyotrophic lateral sclerosis: new clues to understanding an old disease? *Muscle Nerve* 35 712–724 10.1002/mus.20768 17373702

[B186] Nicaise C. Mitrecic D. Demetter P. De Decker R. Authelet M. Boom A. (2009). Impaired blood–brain and blood–spinal cord barriers in mutant SOD1-linked ALS rat. *Brain Res.* 1301 152–162 10.1016/j.brainres.2009.09.018 19748495

[B187] Niebroj-Dobosz I. Janik P. Sokolowska B. Kwiecinski H. (2010). Matrix metalloproteinases and their tissue inhibitors in serum and cerebrospinal fluid of patients with amyotrophic lateral sclerosis. *Eur. J. Neurol.* 17 226–231 10.1111/j.1468-1331.2009.02775.x 19796283

[B188] Niebroj-Dobosz I. Rafalowska J. Fidzianska A. Gadamski R. Grieb P. (2007). Myelin composition of spinal cord in a model of amyotrophic lateral sclerosis (ALS) in SOD1G93A transgenic rats. *Folia Neuropathol.* 45 236–241 18176898

[B189] Nieto-Diaz M. Esteban F. J. Reigada D. Munoz-Galdeano T. Yunta M. Caballero-Lopez M. (2014). MicroRNA dysregulation in spinal cord injury: causes, consequences and therapeutics. *Front. Cell Neurosci.* 8 53 10.3389/fncel.2014.00053 PMC3934005 24701199

[B190] Nikodemova M. Small A. L. Smith S. M. Mitchell G. S. Watters J. J. (2013). Spinal but not cortical microglia acquire an atypical phenotype with high VEGF, galectin-3 and osteopontin, and blunted inflammatory responses in ALS rats. *Neurobiol. Dis.* 10.1016/j.nbd.2013.11.009 [Epub ahead of print] PMC4079765 24269728

[B191] Noda M. Doi Y. Liang J. Kawanokuchi J. Sonobe Y. Takeuchi H. (2011). Fractalkine attenuates excito-neurotoxicity via microglial clearance of damaged neurons and antioxidant enzyme heme oxygenase-1 expression. *J. Biol. Chem.* 286 2308–2319 10.1074/jbc.M110.169839 21071446 PMC3023525

[B192] Offen D. Barhum Y. Melamed E. Embacher N. Schindler C. Ransmayr G. (2009). Spinal cord mRNA profile in patients with ALS: comparison with transgenic mice expressing the human SOD-1 mutant. *J. Mol. Neurosci.* 38 85–93 10.1007/s12031-007-9004-z 18651250

[B193] Ogawa M. Furukawa Y. (2014). A seeded propagation of Cu, Zn-superoxide dismutase aggregates in amyotrophic lateral sclerosis. *Front. Cell. Neurosci.* 8:83 10.3389/fncel.2014.00083 PMC3957682 24672430

[B194] Ohnishi S. Ito H. Suzuki Y. Adachi Y. Wate R. Zhang J. (2009). Intra-bone marrow-bone marrow transplantation slows disease progression and prolongs survival in G93A mutant SOD1 transgenic mice, an animal model mouse for amyotrophic lateral sclerosis. *Brain Res.* 1296 216–224 10.1016/j.brainres.2009.08.012 19686706

[B195] Olah M. Amor S. Brouwer N. Vinet J. Eggen B. Biber K. (2012). Identification of a microglia phenotype supportive of remyelination. *Glia* 60 306–321 10.1002/glia.21266 22072381

[B196] Olivieri G. Baysang G. Meier F. Muller-Spahn F. Stahelin H. B. Brockhaus M. (2001). *N*-acetyl-L-cysteine protects SHSY5Y neuroblastoma cells from oxidative stress and cell cytotoxicity: effects on beta-amyloid secretion and tau phosphorylation. *J. Neurochem.* 76 224–233 10.1046/j.1471-4159.2001.00090.x 11145996

[B197] Otomo A. Pan L. Hadano S. (2012). Dysregulation of the autophagy-endolysosomal system in amyotrophic lateral sclerosis and related motor neuron diseases. *Neurol. Res. Int.* 2012 498428 10.1155/2012/498428 PMC3407648 22852081

[B198] Otto M. Bahn E. Wiltfang J. Boekhoff I. Beuche W. (1998). Decrease of S100 beta protein in serum of patients with amyotrophic lateral sclerosis. *Neurosci. Lett.* 240 171–173 10.1016/S0304-3940(97)00947-6 9502231

[B199] Pan L. Yoshii Y. Otomo A. Ogawa H. Iwasaki Y. Shang H. F. (2012). Different human copper-zinc superoxide dismutase mutants, SOD1G93A and SOD1H46R, exert distinct harmful effects on gross phenotype in mice. *PLoS ONE* 7:e33409 10.1371/journal.pone.0033409 PMC3306410 22438926

[B200] Pankonin M. S. Sohi J. Kamholz J. Loeb J. A. (2009). Differential distribution of neuregulin in human brain and spinal fluid. *Brain Res.* 1258 1–11 10.1016/j.brainres.2008.12.047 19150438

[B201] Papadeas S. T. Maragakis N. J. (2009). Advances in stem cell research for amyotrophic lateral sclerosis. *Curr. Opin. Biotechnol.* 20 545–551 10.1016/j.copbio.2009.09.003 19819686

[B202] Parajuli B. Sonobe Y. Kawanokuchi J. Doi Y. Noda M. Takeuchi H. (2012). GM-CSF increases LPS-induced production of proinflammatory mediators via upregulation of TLR4 and CD14 in murine microglia. *J. Neuroinflammation* 9 268 10.1186/1742-2094-9-268 PMC3565988 23234315

[B203] Parisi C. Arisi I. D’Ambrosi N. Storti A. E. Brandi R. D’Onofrio M. (2013). Dysregulated microRNAs in amyotrophic lateral sclerosis microglia modulate genes linked to neuroinflammation. *Cell Death Dis.* 4 e959 10.1038/cddis.2013.491 PMC3877562 24336079

[B204] Park J. S. Gamboni-Robertson F. He Q. Svetkauskaite D. Kim J. Y. Strassheim D. (2006). High mobility group box 1 protein interacts with multiple Toll-like receptors. *Am. J. Physiol. Cell Physiol.* 290 C917–C924 10.1152/ajpcell.00401.2005 16267105

[B205] Park S. Kim H. T. Yun S. Kim I. S. Lee J. Lee I. S. (2009). Growth factor-expressing human neural progenitor cell grafts protect motor neurons but do not ameliorate motor performance and survival in ALS mice. *Exp. Mol. Med.* 41 487–500 10.3858/emm.2009.41.7.054 19322031 PMC2721146

[B206] Parnes J. R. (1989). Molecular biology and function of CD4 and CD8. *Adv. Immunol.* 44 265–311 10.1016/S0065-2776(08)60644-6 2493728

[B207] Pasquali L. Longone P. Isidoro C. Ruggieri S. Paparelli A. Fornai F. (2009). Autophagy, lithium, and amyotrophic lateral sclerosis. *Muscle Nerve* 40 173–194 10.1002/mus.21423 19609902

[B208] Pehar M. Cassina P. Vargas M. R. Castellanos R. Viera L. Beckman J. S. (2004). Astrocytic production of nerve growth factor in motor neuron apoptosis: implications for amyotrophic lateral sclerosis. *J. Neurochem.* 89 464–473 10.1111/j.1471-4159.2004.02357.x 15056289

[B209] Petanceska S. Canoll P. Devi L. A. (1996). Expression of rat cathepsin S in phagocytic cells. *J. Biol. Chem.* 271 4403–4409 10.1074/jbc.271.8.4403 8626791

[B210] Phani S. Re D. B. Przedborski S. (2012). The role of the innate immune system in ALS. *Front. Pharmacol.* 3:150 10.3389/fphar.2012.00150 PMC3418523 22912616

[B211] Phatnani H. P. Guarnieri P. Friedman B. A. Carrasco M. A. Muratet M. O’Keeffe S. (2013). Intricate interplay between astrocytes and motor neurons in ALS. *Proc. Natl. Acad. Sci. U.S.A.* 110 E756–E765 10.1073/pnas.1222361110 23388633 PMC3581928

[B212] Philips T. Bento-Abreu A. Nonneman A. Haeck W. Staats K. Geelen V. (2013). Oligodendrocyte dysfunction in the pathogenesis of amyotrophic lateral sclerosis. *Brain* 136 471–482 10.1093/brain/aws339 23378219 PMC3572934

[B213] Philips T. Robberecht W. (2011). Neuroinflammation in amyotrophic lateral sclerosis: role of glial activation in motor neuron disease. *Lancet Neurol.* 10 253–263 10.1016/S1474-4422(11)70015-1 21349440

[B214] Ponomarev E. D. Veremeyko T. Weiner H. L. (2013). MicroRNAs are universal regulators of differentiation, activation, and polarization of microglia and macrophages in normal and diseased CNS. *Glia* 61 91–103 10.1002/glia.22363 22653784 PMC3434289

[B215] Poon H. F. Hensley K. Thongboonkerd V. Merchant M. L. Lynn B. C. Pierce W. M. (2005). Redox proteomics analysis of oxidatively modified proteins in G93A-SOD1 transgenic mice – a model of familial amyotrophic lateral sclerosis. *Free Radic. Biol. Med.* 39 453–462 10.1016/j.freeradbiomed.2005.03.030 16043017

[B216] Potolicchio I. Carven G. J. Xu X. Stipp C. Riese R. J. Stern L. J. (2005). Proteomic analysis of microglia-derived exosomes: metabolic role of the aminopeptidase CD13 in neuropeptide catabolism. *J. Immunol.* 175 2237–2243 16081791 10.4049/jimmunol.175.4.2237

[B217] Pottier N. Maurin T. Chevalier B. Puissegur M. P. Lebrigand K. Robbe-Sermesant K. (2009). Identification of keratinocyte growth factor as a target of microRNA-155 in lung fibroblasts: implication in epithelial-mesenchymal interactions. *PLoS ONE* 4:e6718 10.1371/journal.pone.0006718 PMC2726943 19701459

[B218] Pramatarova A. Laganiere J. Roussel J. Brisebois K. Rouleau G. A. (2001). Neuron-specific expression of mutant superoxide dismutase 1 in transgenic mice does not lead to motor impairment. *J. Neurosci.* 21 3369–3374 10.1523/JNEUROSCI.5258-07.2008 11331366 PMC6762496

[B219] Quinn S. R O’Neill L. A. (2011). A trio of microRNAs that control Toll-like receptor signalling. *Int. Immunol.* 23 421–425 10.1093/intimm/dxr034 21652514

[B220] Renton A. E. Chio A. Traynor B. J. (2014). State of play in amyotrophic lateral sclerosis genetics. *Nat. Neurosci.* 17 17–23 10.1038/nn.3584 24369373 PMC4544832

[B221] Renton A. E. Majounie E. Waite A. Simon-Sanchez J. Rollinson S. Gibbs J. R. (2011). A hexanucleotide repeat expansion in C9ORF72 is the cause of chromosome 9p21-linked ALS-FTD. *Neuron* 72 257–268 10.1016/j.neuron.2011.09.010 21944779 PMC3200438

[B222] Rentzos M. Evangelopoulos E. Sereti E. Zouvelou V. Marmara S. Alexakis T. (2012). Alterations of T cell subsets in ALS: a systemic immune activation? *Acta Neurol. Scand.* 125 260–264 10.1111/j.1600-0404.2011.01528.x 21651502

[B223] Reyes N. A. Fisher J. K. Austgen K. Vandenberg S. Huang E. J. Oakes S. A. (2010). Blocking the mitochondrial apoptotic pathway preserves motor neuron viability and function in a mouse model of amyotrophic lateral sclerosis. *J. Clin. Invest.* 120 3673–3679 10.1172/JCI42986 20890041 PMC2947232

[B224] Riboldi G. Nizzardo M. Simone C. Falcone M. Bresolin N. Comi G. P. (2011). ALS genetic modifiers that increase survival of SOD1 mice and are suitable for therapeutic development. *Prog. Neurobiol.* 95 133–148 10.1016/j.pneurobio.2011.07.009 21816207

[B225] Roberts K. Zeineddine R. Corcoran L. Li W. Campbell I. L. Yerbury J. J. (2013). Extracellular aggregated Cu/Zn superoxide dismutase activates microglia to give a cytotoxic phenotype. *Glia* 61 409–419 10.1002/glia.22444 23281114

[B226] Rodier F. Campisi J. (2011). Four faces of cellular senescence. *J. Cell Biol.* 192 547–556 10.1083/jcb.201009094 21321098 PMC3044123

[B227] Rodriguez M. Sabate M. Rodriguez-Sabate C. Morales I. (2013). The role of non-synaptic extracellular glutamate. *Brain Res. Bull.* 93 17–26 10.1016/j.brainresbull.2012.09.018 23149167

[B228] Rojas F. Cortes N. Abarzua S. Dyrda A Van Zundert B. (2014). Astrocytes expressing mutant SOD1 and TDP43 trigger motoneuron death that is mediated via sodium channels and nitroxidative stress. *Front. Cell. Neurosci.* 8:24 10.3389/fncel.2014.00024 PMC3916762 24570655

[B229] Rothstein J. D. (2009). Current hypotheses for the underlying biology of amyotrophic lateral sclerosis. *Ann. Neurol.* 65 (Suppl. 1) S3–S9 10.1002/ana.21543 19191304

[B230] Saba R. Gushue S. Huzarewich R. L. Manguiat K. Medina S. Robertson C. (2012). MicroRNA 146a (miR-146a) is over-expressed during prion disease and modulates the innate immune response and the microglial activation state. *PLoS ONE* 7:e30832 10.1371/journal.pone.0030832 PMC3281888 22363497

[B231] Saijo K. Crotti A. Glass C. K. (2013). Regulation of microglia activation and deactivation by nuclear receptors. *Glia* 61 104–111 10.1002/glia.22423 22987512

[B232] Sanagi T. Yuasa S. Nakamura Y. Suzuki E. Aoki M. Warita H. (2010). Appearance of phagocytic microglia adjacent to motoneurons in spinal cord tissue from a presymptomatic transgenic rat model of amyotrophic lateral sclerosis. *J. Neurosci. Res.* 88 2736–2746 10.1002/jnr.22424 20648658

[B233] Sá-Pereira I. Brites D. Brito M. A. (2012). Neurovascular unit: a focus on pericytes. *Mol. Neurobiol.* 45 327–347 10.1007/s12035-012-8244-2 22371274

[B234] Sargsyan S. A. Blackburn D. J. Barber S. C. Grosskreutz J. De Vos K. J. Monk P. N. (2011). A comparison of in vitro properties of resting SOD1 transgenic microglia reveals evidence of reduced neuroprotective function. *BMC Neurosci.* 12:91 10.1186/1471-2202-12-91 PMC3191510 21943126

[B235] Sau D. De Biasi S. Vitellaro-Zuccarello L. Riso P. Guarnieri S. Porrini M. (2007). Mutation of SOD1 in ALS: a gain of a loss of function. *Hum. Mol. Genet.* 16 1604–1618 10.1093/hmg/ddm110 17504823

[B236] Savaskan N. E. Rocha L. Kotter M. R. Baer A. Lubec G. Van Meeteren L. A. (2007). Autotaxin (NPP-2) in the brain: cell type-specific expression and regulation during development and after neurotrauma. *Cell. Mol. Life Sci.* 64 230–243 10.1007/s00018-006-6412-0 17192809 PMC11136012

[B237] Schiffer D. Cordera S. Cavalla P. Migheli A. (1996). Reactive astrogliosis of the spinal cord in amyotrophic lateral sclerosis. *J. Neurol. Sci.* 139(Suppl.) 27–33 10.1016/0022-510X(96)00073-1 8899654

[B238] Schoser B. G. Blottner D. (1999). Matrix metalloproteinases MMP-2, MMP-7 and MMP-9 in denervated human muscle. *Neuroreport* 10 2795–2797 10.1097/00001756-199909090-00018 10511442

[B239] Shi P. Gal J. Kwinter D. M. Liu X. Zhu H. (2010a). Mitochondrial dysfunction in amyotrophic lateral sclerosis. *Biochim. Biophys. Acta* 1802 45–51 10.1016/j.bbadis.2009.08.012 19715760 PMC2790551

[B240] Shi P. Wei Y. Zhang J. Gal J. Zhu H. (2010b). Mitochondrial dysfunction is a converging point of multiple pathological pathways in amyotrophic lateral sclerosis. *J. Alzheimers. Dis.* 20(Suppl. 2) S311–S324 10.3233/JAD-2010-100366 20463400

[B241] Shobha K. Alladi P. A. Nalini A. Sathyaprabha T. N. Raju T. R. (2010). Exposure to CSF from sporadic amyotrophic lateral sclerosis patients induces morphological transformation of astroglia and enhances GFAP and S100beta expression. *Neurosci. Lett.* 473 56–61 10.1016/j.neulet.2010.02.022 20170712

[B242] Silva S. L. Osório C. Vaz A. R. Barateiro A. Falcão A. S. Silva R. F. (2011). Dynamics of neuron–glia interplay upon exposure to unconjugated bilirubin. *J. Neurochem.* 117 412–424 10.1111/j.1471-4159.2011.07200.x 21275990

[B243] Silva S. L. Vaz A. R. Barateiro A. Falcão A. S. Fernandes A. Brito M. A. (2010). Features of bilirubin-induced reactive microglia: from phagocytosis to inflammation. *Neurobiol. Dis.* 40 663–675 10.1016/j.nbd.2010.08.010 20727973

[B244] Silva S. L. Vaz A. R. Diogenes M. J. Van Rooijen N. Sebastiao A. M. Fernandes A. (2012). Neuritic growth impairment and cell death by unconjugated bilirubin is mediated by NO and glutamate, modulated by microglia, and prevented by glycoursodeoxycholic acid and interleukin-10. *Neuropharmacology* 62 2397–2407 10.1016/j.neuropharm.2012.02.002 22361233

[B245] Sofroniew M. V. (2009). Molecular dissection of reactive astrogliosis and glial scar formation. *Trends Neurosci.* 32 638–647 10.1016/j.tins.2009.08.002 19782411 PMC2787735

[B246] Song F. Chiang P. Wang J. Ravits J. Loeb J. A. (2012). Aberrant neuregulin 1 signalling in amyotrophic lateral sclerosis. *J. Neuropathol. Exp. Neurol.* 71 104–115 10.1097/NEN.0b013e3182423c43 22249457 PMC3270561

[B247] Soon C. P. Crouch P. J. Turner B. J. Mclean C. A. Laughton K. M. Atkin J. D. (2010). Serum matrix metalloproteinase-9 activity is dysregulated with disease progression in the mutant SOD1 transgenic mice. *Neuromuscul. Disord.* 20 260–266 10.1016/j.nmd.2009.11.015 20097566

[B248] Spalloni A. Nutini M. Longone P. (2013). Role of the *N*-methyl-D-aspartate receptors complex in amyotrophic lateral sclerosis. *Biochim. Biophys. Acta* 1832 312–322 10.1016/j.bbadis.2012.11.013 23200922

[B249] Staats K. A. Schonefeldt S. Van Rillaer M. Van Hoecke A. Van Damme P. Robberecht W. (2013). Beta-2 microglobulin is important for disease progression in a murine model for amyotrophic lateral sclerosis. *Front. Cell. Neurosci.* 7:249 10.3389/fncel.2013.00249 PMC3857886 24368896

[B250] Stoorvogel W. (2012). Functional transfer of microRNA by exosomes. *Blood* 119 646–648 10.1182/blood-2011-11-389478 22262739

[B251] Streit W. J. (2006). Microglial senescence: does the brain’s immune system have an expiration date? *Trends Neurosci.* 29 506–510 10.1016/j.tins.2006.07.001 16859761

[B252] Streit W. J. Sammons N. W. Kuhns A. J. Sparks D. L. (2004). Dystrophic microglia in the aging human brain. *Glia* 45 208–212 10.1002/glia.10319 14730714

[B253] Sun D. Zhuang X. Zhang S. Deng Z. B. Grizzle W. Miller D. (2013). Exosomes are endogenous nanoparticles that can deliver biological information between cells. *Adv. Drug Deliv. Rev.* 65 342–347 10.1016/j.addr.2012.07.002 22776312

[B254] Sundaram J. R. Chan E. S. Poore C. P. Pareek T. K. Cheong W. F. Shui G. (2012). Cdk5/p25-induced cytosolic PLA2-mediated lysophosphatidylcholine production regulates neuroinflammation and triggers neurodegeneration. *J. Neurosci.* 32 1020–1034 10.1523/JNEUROSCI.5177-11.2012 22262900 PMC6621136

[B255] Sunnemark D. Eltayeb S. Nilsson M. Wallstrom E. Lassmann H. Olsson T. (2005). CX3CL1 (fractalkine) and CX3CR1 expression in myelin oligodendrocyte glycoprotein-induced experimental autoimmune encephalomyelitis: kinetics and cellular origin. *J. Neuroinflammation* 2 17 10.1186/1742-2094-2-17 PMC1188067 16053521

[B256] Sussmuth S. D. Tumani H. Ecker D. Ludolph A. C. (2003). Amyotrophic lateral sclerosis: disease stage related changes of tau protein and S100 beta in cerebrospinal fluid and creatine kinase in serum. *Neurosci. Lett.* 353 57–60 10.1016/j.neulet.2003.09.018 14642437

[B257] Suzuki M. El-Hage N. Zou S. Hahn Y. K. Sorrell M. E. Sturgill J. L. (2011). Fractalkine/CX3CL1 protects striatal neurons from synergistic morphine and HIV-1 Tat-induced dendritic losses and death. *Mol. Neurodegener.* 6 78 10.1186/1750-1326-6-78 PMC3287119 22093090

[B258] Synofzik M. Fernandez-Santiago R. Maetzler W. Schols L. Andersen P. M. (2010). The human G93A SOD1 phenotype closely resembles sporadic amyotrophic lateral sclerosis. *J. Neurol. Neurosurg. Psychiatry* 81 764–767 10.1136/jnnp.2009.181719 20176600

[B259] Takeuchi H. Jin S. Wang J. Zhang G. Kawanokuchi J. Kuno R. (2006). Tumor necrosis factor-alpha induces neurotoxicity via glutamate release from hemichannels of activated microglia in an autocrine manner. *J. Biol. Chem.* 281 21362–21368 10.1074/jbc.M600504200 16720574

[B260] Taylor D. L. Pirianov G. Holland S. Mcginnity C. J. Norman A. L. Reali C. (2010). Attenuation of proliferation in oligodendrocyte precursor cells by activated microglia. *J. Neurosci. Res.* 88 1632–1644 10.1002/jnr.22335 20091773

[B261] Thams S. Brodin P. Plantman S. Saxelin R. Karre K. Cullheim S. (2009). Classical major histocompatibility complex class I molecules in motoneurons: new actors at the neuromuscular junction. *J. Neurosci.* 29 13503–13515 10.1523/JNEUROSCI.0981-09.2009 19864563 PMC6665011

[B262] Tian L. Ma L. Kaarela T. Li Z. (2012). Neuroimmune crosstalk in the central nervous system and its significance for neurological diseases. *J. Neuroinflammation* 9 155 10.1186/1742-2094-9-155 PMC3410819 22747919

[B263] Tilleux S. Berger J. Hermans E. (2007). Induction of astrogliosis by activated microglia is associated with a down-regulation of metabotropic glutamate receptor 5. *J. Neuroimmunol.* 189 23–30 10.1016/j.jneuroim.2007.06.011 17628702

[B264] Tolosa L. Caraballo-Miralles V. Olmos G. Llado J. (2011). TNF-alpha potentiates glutamate-induced spinal cord motoneuron death via NF-kappaB. *Mol. Cell. Neurosci.* 46 176–186 10.1016/j.mcn.2010.09.001 20849956

[B265] Tovar Y. R. L. B. Santa-Cruz L. D. Tapia R. (2009a). Experimental models for the study of neurodegeneration in amyotrophic lateral sclerosis. *Mol. Neurodegener.* 4 31 10.1186/1750-1326-4-31 PMC2720968 19619317

[B266] Tovar Y. R. L. B. Santa-Cruz L. D. Zepeda A. Tapia R. (2009b). Chronic elevation of extracellular glutamate due to transport blockade is innocuous for spinal motoneurons in vivo. *Neurochem. Int.* 54 186–191 10.1016/j.neuint.2008.09.015 19100799

[B267] Tsou C. L. Haskell C. A. Charo I. F. (2001). Tumor necrosis factor-alpha-converting enzyme mediates the inducible cleavage of fractalkine. *J. Biol. Chem.* 276 44622–44626 10.1074/jbc.M107327200 11571300

[B268] Turner B. J. Talbot K. (2008). Transgenics, toxicity and therapeutics in rodent models of mutant SOD1-mediated familial ALS. *Prog. Neurobiol.* 85 94–134 10.1016/j.pneurobio.2008.01.001 18282652

[B269] Turner M. R. Hardiman O. Benatar M. Brooks B. R. Chio A. De Carvalho M. (2013). Controversies and priorities in amyotrophic lateral sclerosis. *Lancet Neurol.* 12 310–322 10.1016/S1474-4422(13)70036-X 23415570 PMC4565161

[B270] Turola E. Furlan R. Bianco F. Matteoli M. Verderio C. (2012). Microglial microvesicle secretion and intercellular signalling. *Front. Physiol.* 3:149 10.3389/fphys.2012.00149 PMC3357554 22661954

[B271] Uccelli A. Milanese M. Principato M. C. Morando S. Bonifacino T. Vergani L. (2012). Intravenous mesenchymal stem cells improve survival and motor function in experimental amyotrophic lateral sclerosis. *Mol. Med.* 18 794–804 10.2119/molmed.2011.00498 22481270 PMC3409288

[B272] Urushitani M. Sik A. Sakurai T. Nukina N. Takahashi R. Julien J. P. (2006). Chromogranin-mediated secretion of mutant superoxide dismutase proteins linked to amyotrophic lateral sclerosis. *Nat. Neurosci.* 9 108–118 10.1038/nn1603 16369483

[B273] Valadi H. Ekstrom K. Bossios A. Sjostrand M. Lee J. J. Lotvall J. O. (2007). Exosome-mediated transfer of mRNAs and microRNAs is a novel mechanism of genetic exchange between cells. *Nat. Cell Biol.* 9 654–659 10.1038/ncb1596 17486113

[B274] Valori C. F. Brambilla L. Martorana F. Rossi D. (2014). The multifaceted role of glial cells in amyotrophic lateral sclerosis. *Cell. Mol. Life Sci.* 71 287–297 10.1007/s00018-013-1429-7 23912896 PMC11113174

[B275] Van Den Bosch L. (2011). Genetic rodent models of amyotrophic lateral sclerosis. *J. Biomed. Biotechnol.* 2011 348765 10.1155/2011/348765 PMC3022221 21274268

[B276] van Weering H. R. De Jong A. P. De Haas A. H. Biber K. P. Boddeke H. W. (2010). CCL21-induced calcium transients and proliferation in primary mouse astrocytes: CXCR3-dependent and independent responses. *Brain Behav. Immun.* 24 768–775 10.1016/j.bbi.2009.04.007 19401230

[B277] Vaz A. R. Cunha C. Comes C. Fernandes A. Brites D. (2014). Glycoursodeoxycholic acid reduces matrix metalloproteinase-9 and caspase-9 activation in a cellular model of superoxide-dismutase-1 neurodegeneration. *Mol. Neurobiol.* 10.1007/s12035-014-8731-8 [Epub ahead of print] 24848512

[B278] Verbeeck C. Deng Q. Dejesus-Hernandez M. Taylor G. Ceballos-Diaz C. Kocerha J. (2012). Expression of fused in sarcoma mutations in mice recapitulates the neuropathology of FUS proteinopathies and provides insight into disease pathogenesis. *Mol. Neurodegener.* 7 53 10.1186/1750-1326-7-53 PMC3519790 23046583

[B279] Verge G. M. Milligan E. D. Maier S. F. Watkins L. R. Naeve G. S. Foster A. C. (2004). Fractalkine (CX3CL1) and fractalkine receptor (CX3CR1) distribution in spinal cord and dorsal root ganglia under basal and neuropathic pain conditions. *Eur. J. Neurosci.* 20 1150–1160 10.1111/j.1460-9568.2004.03593.x 15341587

[B280] Walter L. Neumann H. (2009). Role of microglia in neuronal degeneration and regeneration. *Semin. Immunopathol.* 31 513–525 10.1007/s00281-009-0180-5 19763574

[B281] Wang L. Pytel P. Feltri M. L. Wrabetz L. Roos R. P. (2012). Selective knockdown of mutant SOD1 in Schwann cells ameliorates disease in G85R mutant SOD1 transgenic mice. *Neurobiol. Dis.* 48 52–57 10.1016/j.nbd.2012.05.014 22668777

[B282] Wegorzewska I. Bell S. Cairns N. J. Miller T. M. Baloh R. H. (2009). TDP-43 mutant transgenic mice develop features of ALS and frontotemporal lobar degeneration. *Proc. Natl. Acad. Sci. U.S.A.* 106 18809–18814 10.1073/pnas.0908767106 19833869 PMC2762420

[B283] Welser J. V. Milner R. (2012). Use of astrocyte–microglial cocultures to examine the regulatory influence of astrocytes on microglial activation. *Methods Mol. Biol.* 814 367–380 10.1007/978-1-61779-452-0_24 22144319

[B284] Wen Z. Xu L. Chen X. Xu W. Yin Z. Gao X. (2013). Autoantibody induction by DNA-containing immune complexes requires HMGB1 with the TLR2/microRNA-155 pathway. *J. Immunol.* 190 5411–5422 10.4049/jimmunol.1203301 23616573

[B285] Weydt P. Yuen E. C. Ransom B. R. Moller T. (2004). Increased cytotoxic potential of microglia from ALS-transgenic mice. *Glia* 48 179–182 10.1002/glia.20062 15378658

[B286] Wilcock D. M. (2012). A changing perspective on the role of neuroinflammation in Alzheimer’s disease. *Int. J. Alzheimers Dis.* 2012 495243 10.1155/2012/495243 PMC3403314 22844636

[B287] Willemen H. L. Huo X. J. Mao-Ying Q. L. Zijlstra J. Heijnen C. J. Kavelaars A. (2012). MicroRNA-124 as a novel treatment for persistent hyperalgesia. *J. Neuroinflammation* 9 143 10.1186/1742-2094-9-143 PMC3418202 22731384

[B288] Willier S. Butt E. Richter G. H. Burdach S. Grunewald T. G. (2011). Defining the role of TRIP6 in cell physiology and cancer. *Biol. Cell* 103 573–591 10.1042/BC20110077 22054418

[B289] Winkler E. A. Sengillo J. D. Sullivan J. S. Henkel J. S. Appel S. H. Zlokovic B. V. (2013). Blood–spinal cord barrier breakdown and pericyte reductions in amyotrophic lateral sclerosis. *Acta Neuropathol.* 125 111–120 10.1007/s00401-012-1039-8 22941226 PMC3535352

[B290] Wong W. T. (2013). Microglial aging in the healthy CNS: phenotypes, drivers, and rejuvenation. *Front. Cell. Neurosci.* 7:22 10.3389/fncel.2013.00022 PMC3595516 23493481

[B291] Xu D. Tahara H. (2013). The role of exosomes and microRNAs in senescence and aging. *Adv. Drug Deliv. Rev.* 65 368–375 10.1016/j.addr.2012.07.010 22820533

[B292] Xu L. Yang B. F. Ai J. (2013). MicroRNA transport: a new way in cell communication. *J. Cell. Physiol.* 228 1713–1719 10.1002/jcp.24344 23460497

[B293] Xu X. Warrington A. E. Bieber A. J. Rodriguez M. (2011). Enhancing CNS repair in neurological disease: challenges arising from neurodegeneration and rewiring of the network. *CNS Drugs* 25 555–573 10.2165/11587830-000000000-00000 21699269 PMC3140701

[B294] Yamanaka K. Boillee S. Roberts E. A. Garcia M. L. Mcalonis-Downes M. Mikse O. R. (2008a). Mutant SOD1 in cell types other than motor neurons and oligodendrocytes accelerates onset of disease in ALS mice. *Proc. Natl. Acad. Sci. U.S.A.* 105 7594–7599 10.1073/pnas.0802556105 18492803 PMC2396671

[B295] Yamanaka K. Chun S. J. Boillee S. Fujimori-Tonou N. Yamashita H. Gutmann D. H. (2008b). Astrocytes as determinants of disease progression in inherited amyotrophic lateral sclerosis. *Nat. Neurosci.* 11 251–253 10.1038/nn2047 18246065 PMC3137510

[B296] Yang C. Wang H. Qiao T. Yang B. Aliaga L. Qiu L. (2014). Partial loss of TDP-43 function causes phenotypes of amyotrophic lateral sclerosis. *Proc. Natl. Acad. Sci. U.S.A.* 111 E1121–E1129 10.1073/pnas.1322641111 24616503 PMC3970502

[B297] Yang W. W. Sidman R. L. Taksir T. V. Treleaven C. M. Fidler J. A. Cheng S. H. (2011). Relationship between neuropathology and disease progression in the SOD1(G93A) ALS mouse. *Exp. Neurol.* 227 287–295 10.1016/j.expneurol.2010.11.019 21145892

[B298] Yi M. H. Zhang E. Kang J. W. Shin Y. N. Byun J. Y. Oh S. H. (2012). Expression of CD200 in alternative activation of microglia following an excitotoxic lesion in the mouse hippocampus. *Brain Res.* 1481 90–96 10.1016/j.brainres.2012.08.053 22975132

[B299] Yin H. Z. Weiss J. H. (2012). Marked synergism between mutant SOD1 and glutamate transport inhibition in the induction of motor neuronal degeneration in spinal cord slice cultures. *Brain Res.* 1448 153–162 10.1016/j.brainres.2012.02.005 22370146 PMC3309159

[B300] Yoshihara T. Ishigaki S. Yamamoto M. Liang Y. Niwa J. Takeuchi H. (2002). Differential expression of inflammation- and apoptosis-related genes in spinal cords of a mutant SOD1 transgenic mouse model of familial amyotrophic lateral sclerosis. *J. Neurochem.* 80 158–167 10.1046/j.0022-3042.2001.00683.x 11796754

[B301] Yoshii Y. Otomo A. Pan L. Ohtsuka M. Hadano S. (2011). Loss of glial fibrillary acidic protein marginally accelerates disease progression in a SOD1(H46R) transgenic mouse model of ALS. *Neurosci. Res.* 70 321–329 10.1016/j.neures.2011.03.006 21453731

[B302] Zhao W. Beers D. R. Appel S. H. (2013). Immune-mediated mechanisms in the pathoprogression of amyotrophic lateral sclerosis. *J. Neuroimmune Pharmacol.* 8 888–899 10.1007/s11481-013-9489-x 23881705 PMC4126425

[B303] Zhao W. Beers D. R. Henkel J. S. Zhang W. Urushitani M. Julien J. P. (2010). Extracellular mutant SOD1 induces microglial-mediated motoneuron injury. *Glia* 58 231–243 10.1002/glia.20919 19672969 PMC2784168

[B304] Zhao W. Beers D. R. Liao B. Henkel J. S. Appel S. H. (2012). Regulatory T lymphocytes from ALS mice suppress microglia and effector T lymphocytes through different cytokine-mediated mechanisms. *Neurobiol. Dis.* 48 418–428 10.1016/j.nbd.2012.07.008 22820142 PMC3897268

[B305] Zhao W. Xie W. Xiao Q. Beers D. R. Appel S. H. (2006). Protective effects of an anti-inflammatory cytokine, interleukin-4, on motoneuron toxicity induced by activated microglia. *J. Neurochem.* 99 1176–1187 10.1111/j.1471-4159.2006.04172.x 17018025

[B306] Zhong Z. Deane R. Ali Z. Parisi M. Shapovalov Y. O’Banion M. K. (2008). ALS-causing SOD1 mutants generate vascular changes prior to motor neuron degeneration. *Nat. Neurosci.* 11 420–422 10.1038/nn2073 18344992 PMC2895310

[B307] Zoccolella S. Beghi E. Palagano G. Fraddosio A. Guerra V. Samarelli V. (2007). Riluzole and amyotrophic lateral sclerosis survival: a population-based study in southern Italy. *Eur. J. Neurol.* 14 262–268 10.1111/j.1468-1331.2006.01575.x 17355545

[B308] Zujovic V. Benavides J. Vige X. Carter C. Taupin V. (2000). Fractalkine modulates TNF-alpha secretion and neurotoxicity induced by microglial activation. *Glia* 29 305–315 10.1002/(SICI)1098-1136(20000215)29:4<305 10652441

